# The Morphological Diversity of Antlion Larvae and Their Closest Relatives over 100 Million Years

**DOI:** 10.3390/insects13070587

**Published:** 2022-06-27

**Authors:** Carolin Haug, Victor Posada Zuluaga, Ana Zippel, Florian Braig, Patrick Müller, Carsten Gröhn, Thomas Weiterschan, Jörg Wunderlich, Gideon T. Haug, Joachim T. Haug

**Affiliations:** 1 Faculty of Biology, Biocenter, Ludwig-Maximilians-Universität München (LMU Munich), Großhaderner Str. 2, 82152 Planegg-Martinsried, Germany; chaug@bio.lmu.de (C.H.); vicposadaz@gmail.com (V.P.Z.); zip-pel@biologie.uni-muenchen.de (A.Z.); braig@biologie.uni-muenchen.de (F.B.); gideon.haug@palaeo-evo-devo.info (G.T.H.); 2 GeoBio-Center at LMU, Richard-Wagner-Str. 10, 80333 München, Germany; 3 Independent Researcher, Kreuzbergstr. 90, 66482 Zweibrücken, Germany; pat14789@web.de; 4 Independent Researcher, Bünebüttler Weg 7, 21509 Glinde, Germany; jcgroehn@t-online.de; 5 Independent Researcher, Forsteler Str. 1, 64739 Höchst im Odenwald, Germany; thomas.weiterschan@web.de; 6 Independent Researcher, Oberer Haeuselbergweg 24, 69493 Hirschberg, Germany; joergwunderlich@t-online.de

**Keywords:** Ascalaphidae, Myrmeleontidae, Myrmeleontiformia, quantitative morphology, Kachin amber, Myanmar

## Abstract

**Simple Summary:**

The larvae of owlflies and antlions (here shortly embraced by the term “owllions”) are ambush predators. Their mouthparts are transformed into teeth-bearing stylets and used for catching prey and sucking, which is characteristic for neuropteran larvae. Here we used the morphology of the stylets and the head capsules of a large number of extant and fossil larvae as a proxy for the morphological diversity over time. The created dataset comprises outlines of stylets and head capsules of specimens from the literature, collections, databases and the herein described and depicted 38 fossil ones. Fossils in the whole dataset come from deposits with an age of about 20, 40, and 100 million years (Miocene, Eocene, and Cretaceous, respectively). In addition to the shape analysis of the outlines from the dataset, we conducted a statistical analysis as well. Eocene and Miocene samples did not result in a clear output, but Cretaceous samples allowed for some conclusions: The morphological diversity of owllion larvae increased over time, even though some morphologies of Cretaceous larvae went extinct.

**Abstract:**

Among lacewings (Neuroptera), representatives of the groups Ascalaphidae (owlflies) and Myrmeleontidae (antlions) are likely the most widely known ones. The exact taxonomic status of the two groups remains currently unclear, each may in fact be nested in the other group. Herein, we refer to the group including representatives of both with the neutral term “owllion”. Owllion larvae are voracious ambush hunters. They are not only known in the extant fauna, but also from the fossil record. We report here new findings of a fossil owlfly larva from Eocene Baltic amber, as well as several owlfly-like larvae from Cretaceous Kachin amber, Myanmar. Based on these fossils, combined with numerous fossil and extant specimens from the literature, collections, and databases, we compared the morphological diversity of the head and mouthpart shapes of the larvae of owllions in the extant fauna with that of owllion-like larvae from three time slices: about 100 million years ago (Cretaceous), about 40 million years ago (Eocene), and about 20 million years ago (Miocene). The comparison reveals that the samples from the Eocene and Miocene are too small for a reliable evaluation. Yet, the Cretaceous larvae allow for some conclusions: (1) the larval morphological diversity of owllion larvae increased over time, indicating a post-Cretaceous diversification; (2) certain morphologies disappeared after the Cretaceous, most likely representing ecological roles that are no longer present nowadays. In comparison, other closely related lineages, e.g., silky lacewings or split-footed lacewings, underwent more drastic losses after the Cretaceous and no subsequent diversifications.

## 1. Introduction

Human society has begun to recognise a major challenge: human-induced climate change, loss of habitats, excessive use of pesticides, and general human interference together have caused a tremendous loss in biodiversity in the last 50 years [1]. Estimates in the last decade indicate a 41% decline in populations of Insecta; about a third of sampled species of this group have been given the status “threatened” [2], overall popularising the expression “insect decline” (e.g., [3,4,5,6,7,8]). This situation affects many ecosystems, for example, due to a loss of key species such as native pollinators, which reduces agricultural output and is hence also an economic issue [9,10].

In face of this situation, recognising which groups are being most affected by the occurring changes is essential in order to coordinate an effective plan to stop further losses of biodiversity. Unfortunately, our estimates of biodiversity in Insecta have traditionally been biased due to a collecting preference in favour of adults (cf. [11]) and a critical undersampling of other developmental stages. Yet, for especially larvae (for challenges of the term, see [12,13,14,15]), which can differ entirely in their ecological function from the adult, are particularly long-lived in many groups and may have critical ecosystem functions (e.g., [16,17]). The focus of sampling adults seems partly due to their easier accessibility, and possibly also due to the taxonomic focus on characteristics only present in the adults.

The strong ecological differentiation between larvae and adults is especially expressed in representatives of the group Holometabola (e.g., [18,19]), including beetles, wasps, moths, and flies. A smaller, less species-rich ingroup of Holometabola is Neuroptera, the group of lacewings [20,21,22]. Within Neuroptera, which are most famous for their larvae and represent a large share of the overall species richness of lacewings, are antlions (traditionally Myrmeleontidae) and “owlflies” (although owllacewings would be the less ambiguous term; traditionally Ascalaphidae). For a long time, Ascalaphidae and Myrmeleontidae have been considered sister groups (e.g., [22]). Yet, newer phylogenetic analyses have challenged this view. The group traditionally termed Ascalaphidae has in some analyses been resolved as being an ingroup of Myrmeleontidae (i.e., Ascalaphidae being nested within Myrmeleontidae [23] and renamed Ascalaphinae [24]). Alternatively, Myrmeleontidae has been resolved as being an ingroup of Ascalaphidae (e.g., [25,26]). The monophyly of a combined group including all representatives traditionally considered Myrmeleontidae and Ascalaphidae seems to not have been challenged.

Larvae of this combined group are ambush predators, using their prominent mouthparts to catch prey [27,28,29,30,31]. Each upper jaw (mandible) and corresponding lower jaw (maxilla) form a stylet, with which prey such as ants is pierced [22,32,33,34]. Some antlion larvae are famous for being pit-builders (e.g., [35,36,37,38]), yet overall the larval ecology and behaviour of other representatives remain relatively unexplored [39,40,41]. Corresponding adults are aerial predators or consume pollen and nectar [33,42,43]. 

Neuroptera is an interesting case to study in the context of biodiversity loss over a long time, as the group is generally understood to have been more diverse in the past and having had declined to a smaller diversity in the extant fauna (e.g., [22,44,45]). With this, Neuroptera offers us an opportunity to study such losses in the past to improve our understanding of similar phenomena in the extant fauna. 

The impression of a more diverse lacewing fauna in the past is provided by numerous fossils, especially from the Mesozoic (e.g., [46,47,48,49,50,51,52,53,54,55,56]), but also from the Cenozoic era (e.g., [57,58,59,60,61,62]). These fossils include numerous different types of lacewing larvae (e.g., [26,47,50,52,62,63,64,65,66,67,68,69,70,71,72,73,74,75,76,77,78,79,80,81,82,83,84,85,86,87]), among them being also larvae that have been interpreted as representatives of the combined group including Myrmeleontidae and Ascalaphidae [47,50,52,57,62,86]. Some of these larvae have more precisely been interpreted as offshoots of the direct evolutionary lineage towards the combined group [50]. Such larvae, on a first glance, strongly resemble the larvae of Ascalaphidae [47,52].

For other ingroups of Neuroptera, quantitative morphological comparison has identified losses of larval diversity over the last 100 million years [75,82], supporting a general loss of diversity within these groups. Yet, some of such studies have remained inconclusive as to whether there was a loss of diversity or a shift (for example into other habitats), which would have partly balanced a certain loss in one lineage by a diversification in another one [78,79,81]. In few instances, even a slight increase of morphological larval diversity could be observed [81,83]. 

Here we quantitatively compared the larval morphological diversity of the combined group, including samples that are traditionally considered Myrmeleontidae and Ascalaphidae. The study is based on the shape of the head and mouthparts for fossils from three different time slices and the extant fauna. We report new specimens from Eocene Baltic amber and Cretaceous Kachin amber, in Myanmar. We furthermore compared the results to those of other lineages within Neuroptera. 

## 2. Materials and Methods

### 2.1. Material

Thirty-eight fossil specimens of lacewing larvae were directly studied. The specimens come from different collections: the University of Rennes, France (IGR.ARC), the Palaeo-Evo-Devo Research Group Collection of Arthropods, Ludwig-Maximilians-Universita“t Mu”nchen, Germany (PED), and from the collections of some of the authors, namely C.G. (CCGG), P.M. (BUB), J.W. (CJW F), and T.W. (Weiterschan BuB). All the specimens were legally obtained and are accessible for scientific studies. The specimens from the PED collection were legally purchased via the internet platform ebay.com, from the traders burmitefossil, burmite-miner, burmite-researcher, holding_history, and mi2leon. The dataset for the quantitative analysis was further amended by fossil specimens from the literature. For details on the specimens, see Supplementary File S1.

In addition, 243 extant larvae traditionally interpreted as representatives of Myrmeleontidae (173 specimens) and Ascalaphidae (70 specimens) were investigated. Of these, 101 were directly studied in two museum collections: the Zoologische Staatsammlung München (ZSM) and the Leibniz-Institut zur Analyse des Biodiversitätswandels–Hamburg site (LIB, formerly Centrum für Naturkunde/CeNak/ZMH). The remaining specimens were based on literature data and on databases and image repositories. Details of the specimens are provided in Supplementary File S1; for additional references, see Supplementary File S2.

### 2.2. Documentation Methods

Directly investigated specimens were documented using either a Keyence VHX-6000 digital microscope or a super-macro-photography set-up. The latter included a Canon EOS 650D, with a Canon MP-E 65 mm macrolens. Lighting was provided by either a twin-macro flash (Yongnuo YN24EX E-TTL) or a set of two separate flashes (Yongnuo Digital Speedlite YN560EX II). Flashes and lenses were equipped with polarizers to achieve cross-polarized light. 

Extant specimens were documented in 70% ethanol (original storage liquid) or under dry conditions, for specimens mounted on needles. Amber specimens were immersed with glycerol or water and a cover slip was put on top. Specimens documented on the VHX microscope were documented with ring light and cross-polarized coaxial light in front of a black and white background. The images providing the best details were further used (for details, see [88]). 

All images were recorded as composite images [89], and images on the VHX microscope were additionally recorded as HDR [88]. Further processing was performed in Adobe Photoshop CS2. 

### 2.3. Digital Drawings

The head capsule and stylets of all individuals were outlined through vector-drawing programs (Inkscape 1.1, Adobe Illustrator CS2). The outline of the better-accessible half was drawn. The stylet was artificially rotated to be straight; the innermost distal point and the innermost proximal point were oriented to form a line parallel to the anterior–posterior midline [79]. Then, the half object was mirrored.

### 2.4. Shape Analysis

In the quantitative analysis, in total, 300 specimens could be included: 57 fossil larvae from three different time periods (9 Miocene, 2 Eocene, 46 Cretaceous) and 243 extant larvae. An elliptic Fourier analysis was performed using the SHAPE software package [90] in order to convert the outlines to quantifiable data. Similar analyses were performed, e.g., in [75,78,79]. 

### 2.5. Statistical Analysis

The statistical analysis was conducted and visualized using the R-statistics envi-ronment, ver. 4.1.0, Vienna, Austria [91], using the packages *dispRity*, ver. 1.6.0 [92], *ggplot2*, ver. 3.3.5 [93], *reshape2*, ver. 1.4.4 [94], *vegan*, ver.2.5-7 [95], and *RColorBrewer*, ver. 1.1-2 [96].

The morphospace resulting from the PCA matrix of the shape analysis was investigated using multidimensional analysis [97]. It was statistically tested whether the occupied areas of larvae from different time periods within the morphospace were significantly different. Additionally, we tested the size and position of the occupied morphospace of extinct and extant representatives. The change in position was measured by calculating the average displacement of individuals, and the occupied size was measured by the sum of variances of individual groups [97]. Differences between groups were tested using a PERMANOVA test and a series of bootstrapped, Bonferroni-corrected random sampling tests [97,98]. Since the sample sizes of individual groups varied strongly, we used the bootstrapping function and rare faction for sample size correction as provided by the *dispRity* package. 

### 2.6. Note on Terminology

As pointed out, the taxonomy of the group in focus is currently in flux. At least one of the two traditional names of Myrmeleontidae and Ascalaphidae will be considered invalid and changed according to the Linnean rank system; for example, Machado et al. [24] suggested that Ascalaphidae needs to be changed to Ascalaphinae. The analysis of Badano et al. [26] would in principle indicate the opposite (Myrmeleontidae as an ingroup of Ascalaphidae). This conflict indicates a general weakness of any ranked taxonomic approach (see also, e.g., [99,100,101]). While Aves and Reptilia have long been considered as separate classes, a more recent view now simply considers Aves as an ingroup of Reptilia [102], which preserves both names. In the current situation, we see ourselves unable to provide a solution for the Ascalaphidae–Myrmeleontidae problem. To make clear that we cannot decide for the one or other taxonomic view, we will use here a simple expression for the entirety of all representatives that have been traditionally considered as Myrmeleontidae and Ascalaphidae. We will use here the expression “owllions” for this purpose. While this may be unsatisfying from a taxonomic point of view, it will be easier to read in the text than “representatives traditionally considered Myrmeleontidae and Ascalaphidae”. Furthermore, “owllions” will be easily understood by all readers who are roughly familiar with the group and it also makes a reference to the unclear situation.

For the morphological structures, we use neutral terminology to allow for non-specialists to follow our descriptions. Examples of used terms are as follows: leg 3 = hindleg; locomotory appendages = (walking) legs; stemmata = larval eyes.

## 3. Results

### 3.1. Short Descriptions of New Specimens

**Figure 1:**
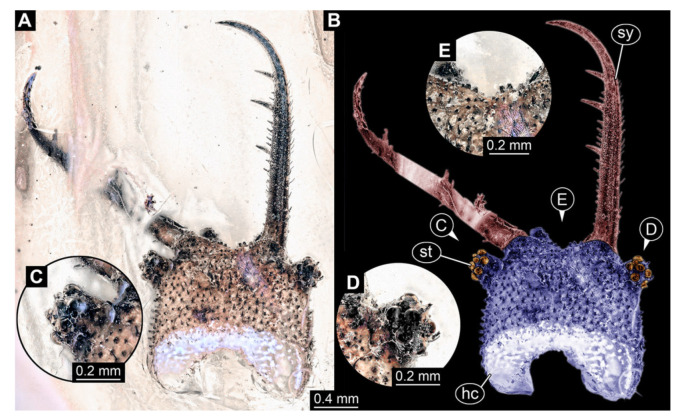
Fossil owllion larva, specimen 3102 (CCGG 2525), Eocene Baltic amber. (**A**) Overview, dorsal. (**B**) Color-marked version of (**A**). (**C**) Close-up on stemmata, left side. (**D**) Close-up on stemmata, right side. (**E**) Close-up on antero-median region of head capsule. Abbreviations: hc = head capsule; st = stemmata; sy = stylet.

**Figure 2:**
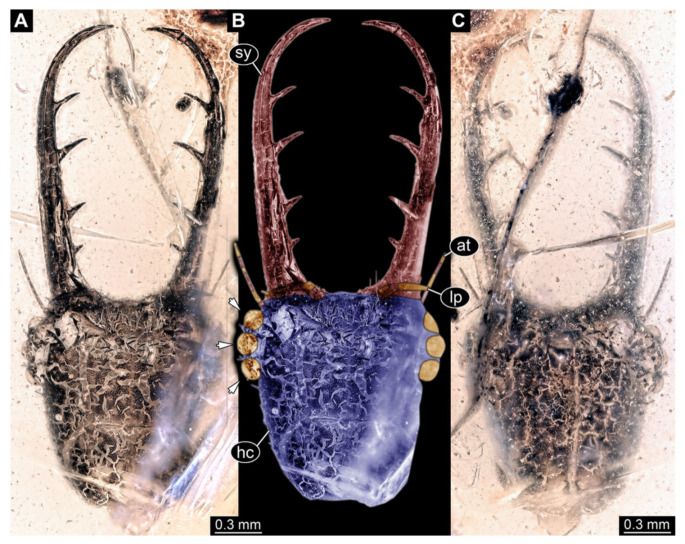
Fossil owllion larva, specimen 3213 (IGR.ARC-236.3), Cretaceous Charentese amber, France. (**A**) Overview, ventral. (**B**) Color-marked version of (**A**); arrows indicate individual stemmata. (**C**) Overview, dorsal. Abbreviations: at = antenna; hc = head capsule; lp = labial palp; sy = stylet.

**Figure 3:**
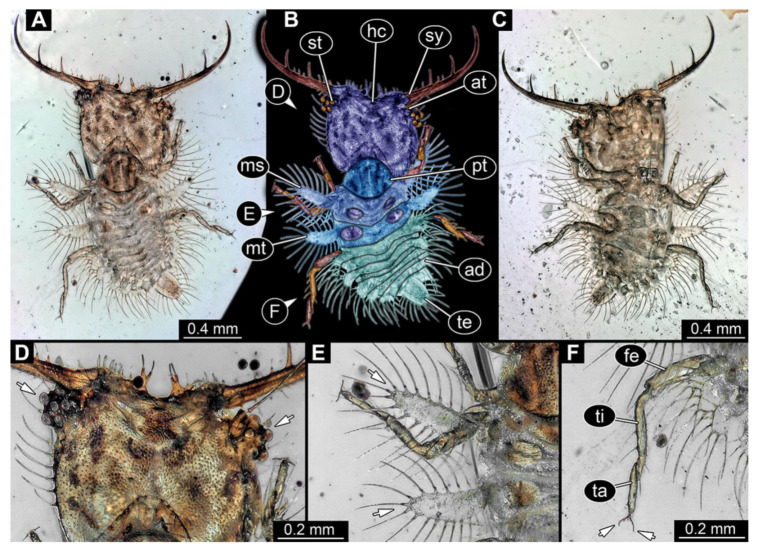
Fossil owllion larva, specimen 3214 (BUB 1430), Cretaceous Kachin amber, Myanmar. (**A**) Overview, dorsal. (**B**) Color-marked version of (**A**). (**C**) Overview, ventral. (**D**) Close-up on head capsule; arrows indicate fields of stemmata. (**E**) Close up on dorsal processes (arrows). (**F**) Close-up on locomotory appendages with claws (arrows). Abbreviations: ad = abdomen; at = antenna; fe = femur; hc = head capsule; ms = mesothorax; mt = metathorax; pt = prothorax; st = stemmata; sy = stylet; ta = tarsus; te = trunk end; ti = tibia.

**Figure 4:**
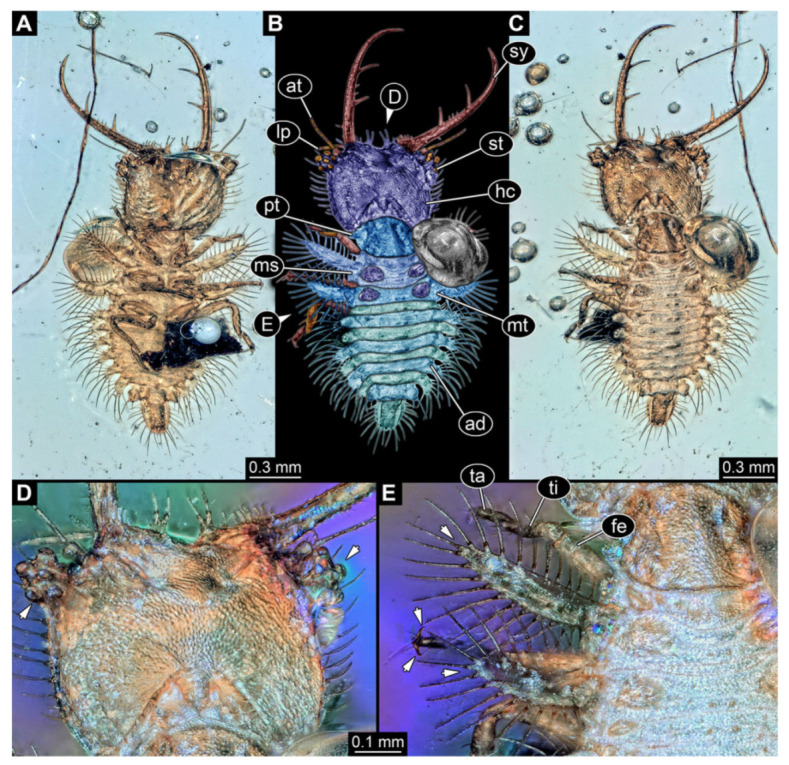
Fossil owllion larva, specimen 3215 (BUB 2537), Cretaceous Kachin amber, Myanmar. (**A**) Overview, ventral. (**B**) Color-marked version of (**C**). (**C**) Overview, dorsal. (**D**) Close-up on head capsule with fields of stemmata (arrows). (**E**) Close up on anterior trunk region with locomotory appendages with distal claws (two middle arrows) and dorsal processes (upper and lower arrow). Abbreviations: ad = abdomen; at = antenna; fe = femur; hc = head capsule; lp = labial palp; ms = mesothorax; mt = metathorax; pt = prothorax; st = stemmata; sy = stylet; ta = tarsus; ti = tibia.

**Figure 5:**
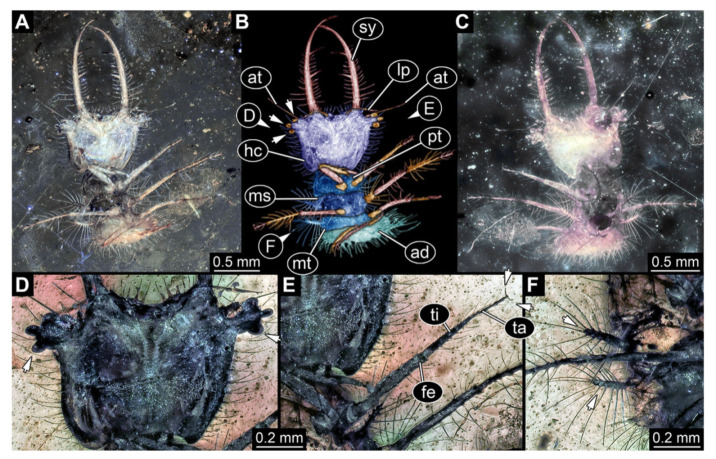
Fossil owllion larva, specimen 3216 (BUB 3343), Cretaceous Kachin amber, Myanmar. (**A**) Overview, ventral. (**B**) Color-marked version of (**A**); arrows mark stemmata. (**C**) Overview, dorsal. (**D**) Close-up on head capsule with fields of stemmata (arrows). (**E**) Close-up on locomotory appendages with distal claws (arrows). (**F**) Close up on dorsal processes (arrows). Abbreviations: ad = abdomen; at = antenna; fe = femur; hc = head capsule; lp = labial palp; ms = mesothorax; mt = metathorax; pt = prothorax; sy = stylet; ta = tarsus; ti = tibia.

**Figure 6:**
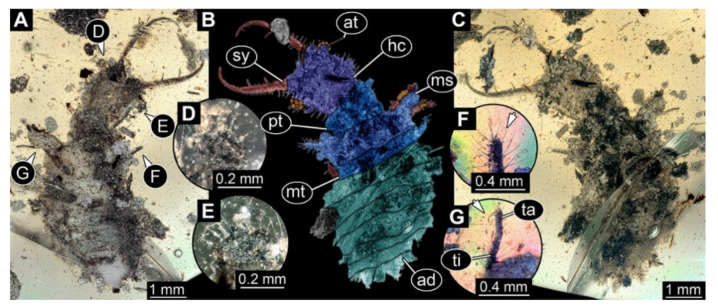
Fossil owllion larva, specimen 3217 (BUB 3348), Cretaceous Kachin amber, Myanmar. (**A**) Overview, ventral. (**B**) Color-marked version of (**C**). (**C**) Overview, dorsal. (**D**,**E**) Close-ups of fields of stemmata. (**F**) Close-up on dorsal process (arrow). (**G**) Close-up on locomotory appendage with distal claws (arrow). Abbreviations: ad = abdomen; at = antenna; hc = head capsule; ms = mesothorax; mt = metathorax; pt = prothorax; sy = stylet; ta = tarsus; ti = tibia.

**Figure 7:**
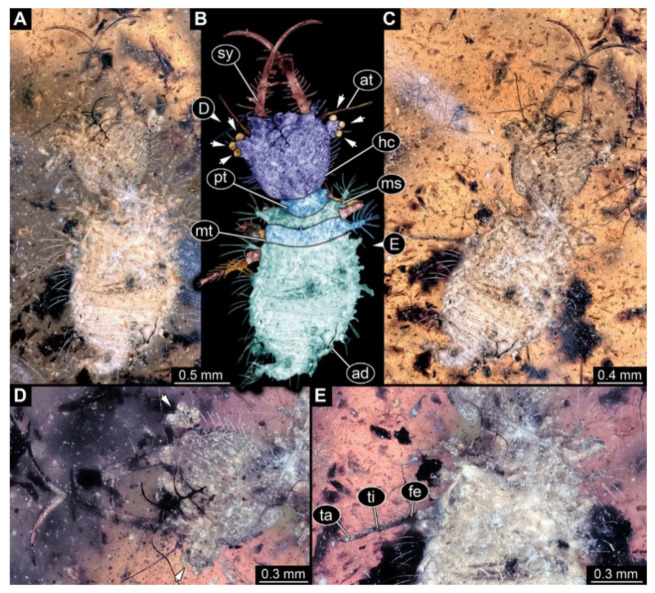
Fossil owllion larva, specimen 3218 (BUB 3378), Cretaceous Kachin amber, Myanmar. (**A**) Overview, dorsal, black background. (**B**) Color-marked version of (**A**). (**C**) Overview, dorsal, white background. (**D**) Close-up on head capsule with fields of stemmata (arrows). (**E**) Close-up on locomotory appendage. Abbreviations: ad = abdomen; at = antenna; fe = femur; hc = head capsule; ms = mesothorax; mt = metathorax; pt = prothorax; sy = stylet; ta = tarsus; ti = tibia.

**Figure 8:**
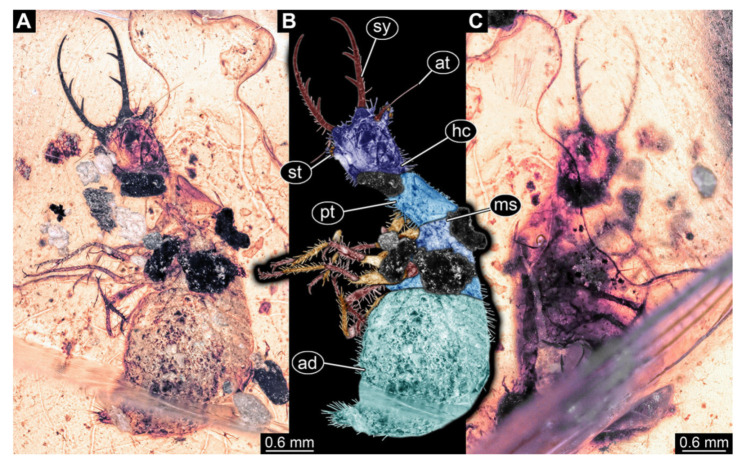
Fossil owllion larva, specimen 3219 (BUB 3380), Cretaceous Kachin amber, Myanmar. (**A**) Overview, oblique dorsal. (**B**) Color-marked version of (**A**). (**C**) Overview, oblique ventral. Abbreviations: ad = abdomen; at = antenna; hc = head capsule; ms = mesothorax; pt = prothorax; st = stemmata; sy = stylet.

**Figure 9:**
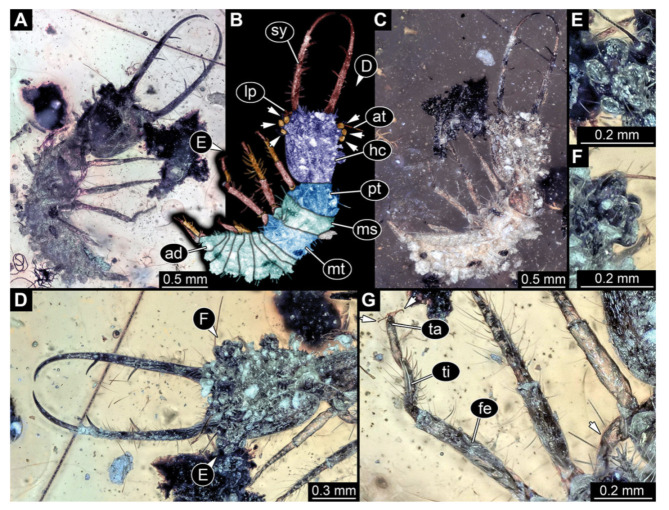
Fossil owllion larva, specimen 3221 (BUB unnumbered), Cretaceous Kachin amber, Myanmar. (**A**) Overview, oblique ventral. (**B**) Color-marked version of (**C**); arrows mark stemmata. (**C**) Overview, oblique dorsal. (**D**) Close-up on head capsule. (**E**,**F**) Close-ups on fields of stemmata. (**G**) Close-up on locomotory appendage, with claws (left arrows) and dorsal process (right arrow). Abbreviations: ad = abdomen; at = antenna; fe = femur; hc = head capsule; lp = labial palp; ms = mesothorax; mt = metathorax; pt = prothorax; sy = stylet; ta = tarsus; ti = tibia.

**Figure 10:**
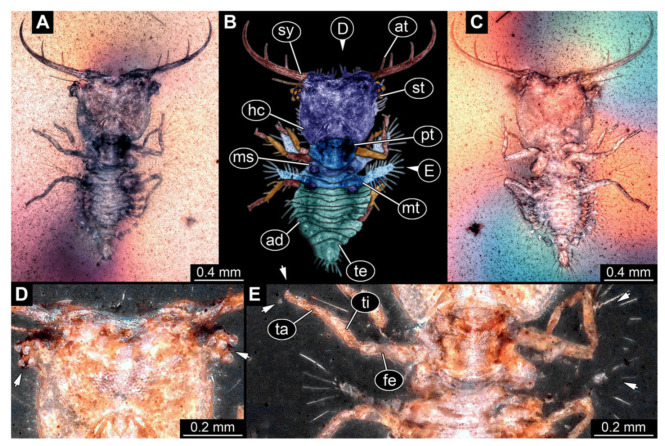
Fossil owllion larva, specimen 3222 (BUB 3062), Cretaceous Kachin amber, Myanmar. (**A**) Overview, dorsal. (**B**) Color-marked version of (**A**). (**C**) Overview, ventral. (**D**) Close-up on head capsule with fields of stemmata (arrows). (**E**) Close-up on anterior trunk with locomotory appendages with claws (left arrows) and dorsal processes (right arrows). Abbreviations: ad = abdomen; at = antenna; fe = femur; hc = head capsule; ms = mesothorax; mt = metathorax; pt = prothorax; st = stemmata; sy = stylet; ta = tarsus; te = trunk end; ti = tibia.

**Figure 11:**
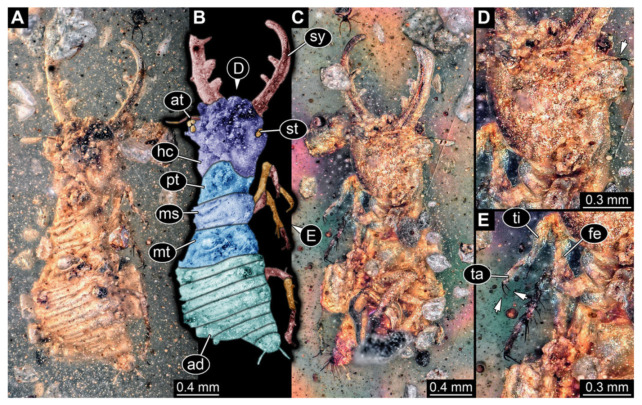
Fossil owllion larva, specimen 3223 (BUB 3063), Cretaceous Kachin amber, Myanmar. (**A**) Overview, dorsal. (**B**) Color-marked version of (**A**). (**C**) Overview, ventral. (**D**) Close-up on head capsule with field of stemmata (arrow). (**E**) Close-up on anterior trunk with locomotory appendages with claws (arrows). Abbreviations: ad = abdomen; at = antenna; fe = femur; hc = head capsule; ms = mesothorax; mt = metathorax; pt = prothorax; st = stemmata; sy = stylet; ta = tarsus; ti = tibia.

In total, 38 fossil specimens of owllion larvae are shortly described here:

1. Specimen 3102 (CCGG 2525), preserved in Eocene Baltic amber. Only the head preserved. Specimen well accessible in dorsal view, with the left stylet partially obscured (Figure 1A,B). Head capsule roughly square-shaped in dorsal view; stylets longer than head capsule (Figure 1A,B). Stemmata on prominent protrusions on each side of head capsule (Figure 1C,D). Each stylet has three major teeth and numerous smaller teeth or robust setae (Figure 1A,B). Anterior rim of head medially with a pair of larger tubercles on each side, close to the midline (Figure 1E). No antenna or palp apparent. Preserved part of specimen is 3.40 mm.

2. Specimen 3213 (IGR.ARC-236.3), preserved in Cretaceous Charentese amber, France. Specimen was already reported in Wang et al. [47] (Figure 3E p. 4), but re-figured here to add some details. Only the head is preserved. Specimen well accessible in ventral (Figure 2A,B) and dorsal view (Figure 2C). Head capsule roughly rectangular in dorsal view, longer than wide; stylets longer than head capsule (Figure 2A–C). Stemmata on slight elevations on each side of head capsule (Figure 2A–C). Antennae small, very slender, far lateral, slightly anterior to stemmata. Each stylet bearing four major teeth, but no smaller teeth or robust setae. Short robust labial palps, with three elements each, antero-lateral, close to stylets. Preserved part of specimen is 3.14 mm.

3. Specimen 3214 (BUB 1430), preserved in Cretaceous Kachin amber, Myanmar. Specimen very complete, well accessible in dorsal (Figure 3A,B) and ventral view (Figure 3C). Head capsule roughly square-shaped in dorsal view, only slightly wider than long; stylets longer than head capsule (Figure 3A–C). Anterior rim with deeply forked spine, one on each side of midline (Figure 3D). Stemmata on prominent protrusions on each side of head capsule (Figure 3D). Antennae small, very slender, far lateral, slightly anterior to stemmata. Each stylet has two major teeth and few smaller teeth or robust setae (Figure 3A–C). No palp apparent. Pronotum cap-like. Mesothorax and metathorax with very prominent dorso-lateral protrusions bearing numerous setae (Figure 3E). Thorax segments ventrally with locomotory appendages. Legs distally with three prominent elements (femur, tibia, tarsus), with the tibia and tarsus well separated, also seen in leg three (Figure 3F). Distal tarsus with pair of claws, no empodium apparent. Anterior abdomen segments also with protrusions, but smaller ones. Trunk end is elongate, longer than wide in dorsal view, with numerous setae. Total length of specimen is 2.15 mm.

4. Specimen 3215 (BUB 2537), preserved in Cretaceous Kachin amber, Myanmar. Specimen is well accessible in ventral view (Figure 4A), dorsally partially concealed by air bubble (Figure 4B,C). Head capsule roughly square-shaped in dorsal view, only slightly wider than long; stylets longer than head capsule (Figure 4A–C). Anterior rim with deeply forked spine, one on each side of midline (Figure 4D). Stemmata on prominent protrusions on each side of head capsule (Figure 4D). Antennae small, very slender, far lateral, slightly anterior to stemmata. Each stylet has two major teeth and few smaller teeth or robust setae (Figure 4A–C). Labial palp is short, robust. Pronotum cap-like. Mesothorax and metathorax with very prominent dorso-lateral protrusions bearing numerous setae (Figure 4E). Thorax segments ventrally with locomotory appendages. Legs distally with three prominent elements (femur, tibia, tarsus), with the tibia and tarsus well separated (Figure 4E). Distal tarsus with a pair of claws, no empodium apparent. Anterior abdomen segments also with protrusions, but smaller ones. Trunk end is elongated, longer than wide in dorsal view, with numerous setae. Total length of specimen is 2.42 mm.

5. Specimen 3216 (BUB 3343), preserved in Cretaceous Kachin amber, Myanmar. Anterior region of specimen is well accessible in ventral (Figure 5A,B) and dorsal view (Figure 5C); posterior part of body folded forward, not well accessible. Head capsule roughly square-shaped in dorsal view, only slightly wider than long; stylets longer than head capsule (Figure 5A–C). Anterior rim with simple setae (Figure 5D). Stemmata on prominent protrusions on each side of head capsule (Figure 5D). Antennae small, very slender, far lateral, slightly anterior to stemmata. Each stylet has two major teeth and few smaller teeth or robust setae (Figure 5A–C). Labial palp is short, robust. Pronotum cap-like. Mesothorax and metathorax with very prominent dorso-lateral protrusions bearing numerous setae (Figure 5F). Thorax segments ventrally with locomotory appendages. Legs distally with three prominent elements (femur, tibia, tarsus), with the tibia and tarsus well separated (Figure 5E). Distal tarsus with a pair of claws, no empodium apparent. Anterior abdomen segments also with protrusions, but smaller ones. Trunk end is elongated, longer than wide in dorsal view, with numerous setae. Total length of specimen is 2.60 mm.

6. Specimen 3217 (BUB 3348), preserved in Cretaceous Kachin amber, Myanmar. Specimen is well accessible in ventral (Figure 6A) and dorsal view (Figure 6B,C), yet partly concealed by debris, apparently attached to body forming camouflaging cloak. Head capsule roughly square-shaped in dorsal view; stylets longer than head capsule (Figure 6A–C). Stemmata on prominent protrusions on each side of head capsule (Figure 6D,E). Antennae small, very slender, far lateral, slightly anterior to stemmata. Each stylet has two major teeth and few smaller teeth or robust setae (Figure 6A–C). No palp apparent. Pronotum cap-like. Mesothorax and metathorax with prominent dorso-lateral protrusions bearing numerous setae (Figure 6F). Thorax segments ventrally with locomotory appendages. Legs distally with two prominent elements (tibia, tarsus), with the tibia and tarsus well separated (Figure 6G). Distal tarsus with a pair of claws, no empodium apparent. Details of abdomen not well accessible due to debris. Total length of specimen is 9.00 mm.

7. Specimen 3218 (BUB 3378), preserved in Cretaceous Kachin amber, Myanmar. Specimen is well accessible in dorsal view (Figure 7A–C). Head capsule roughly trapezium-shaped in dorsal view, anterior wider than posterior; stylets longer than head capsule (Figure 7D). Stemmata on prominent protrusions on each side of head capsule (Figure 7D). Antennae small, very slender, far lateral, slightly anterior to stemmata. Each stylet has two major teeth and few smaller teeth or robust setae (Figure 7D). No palp apparent. Pronotum cap-like. Mesothorax and metathorax with prominent dorso-lateral protrusions bearing numerous setae (Figure 7B). Thorax segments ventrally with locomotory appendages. Legs distally with two prominent elements (tibia, tarsus), with the tibia and tarsus well separated (Figure 7E). Anterior abdomen segments also with protrusions, but smaller ones. Trunk end is elongated, longer than wide in dorsal view, with numerous setae. Total length of specimen is 3.26 mm.

8. Specimen 3219 (BUB 3380), preserved in Cretaceous Kachin amber, Myanmar. Specimen is accessible in oblique dorsal (Figure 8A,B) and oblique ventral view (Figure 8C), but partly concealed by debris. Head capsule roughly trapezium-shaped in dorsal view, anterior slightly wider than posterior; stylets longer than head capsule (Figure 8). Stemmata on prominent protrusions on each side of head capsule (Figure 8). Antennae small, very slender, far lateral, slightly anterior to stemmata. Each stylet has two major teeth and few smaller teeth or robust setae (Figure 8). No palp apparent. Pronotum cap-like. Mesothorax and metathorax largely concealed (Figure 8A,B). Thorax segments ventrally with locomotory appendages. Legs distally with two prominent elements (tibia, tarsus), with the tibia and tarsus well separated (Figure 8B). Anterior abdomen segments without apparent protrusions. Trunk end with numerous setae. Total length of specimen is 6.00 mm.

9. Specimen 3221 (BUB unnumbered), preserved in Cretaceous Kachin amber, Myanmar. Specimen is well accessible in oblique ventral (Figure 9A) and oblique dorsal view (Figure 9B,C). Head capsule roughly trapezium-shaped in dorsal view, anterior wider than posterior; stylets longer than head capsule (Figure 9D). Stemmata on prominent protrusions on each side of head capsule (Figure 9E,F). Antennae small, very slender, far lateral, slightly anterior to stemmata. Each stylet has two major teeth and few smaller teeth or robust setae (Figure 9D). No palp apparent. Pronotum cap-like. Mesothorax and metathorax bearing numerous setae (Figure 9A–C). Thorax segments ventrally with locomotory appendages. Legs distally with two prominent elements (tibia, tarsus), with the tibia and tarsus well separated (Figure 9G). Anterior abdomen segments also with numerous setae. Trunk end is elongated, longer than wide in dorsal view, with numerous setae. Total length of specimen is 3.6 mm.

10. Specimen 3222 (BUB 3062), preserved in Cretaceous Kachin amber, Myanmar. Specimen is well accessible in dorsal (Figure 10A,B) and ventral view (Figure 10C). Head capsule roughly square-shaped in dorsal view, only slightly wider than long; stylets longer than head capsule (Figure 10A–C). Anterior rim with deeply forked spine, one on each side of midline (Figure 10A,B). Stemmata on prominent protrusions on each side of head capsule (Figure 10D). Antennae small, very slender, far lateral, slightly anterior to stemmata. Each stylet has two major teeth and few smaller teeth or robust setae (Figure 10A–C). No palps apparent. Pronotum cap-like. Mesothorax and metathorax with very prominent dorso-lateral protrusions bearing numerous setae (Figure 10A–C). Thorax segments ventrally with locomotory appendages. Legs distally with three prominent elements (femur, tibia, tarsus), with the tibia and tarsus well separated (Figure 10E). Distal tarsus with a pair of claws, no empodium apparent. Anterior abdomen segments also with protrusions, but smaller ones. Trunk end is elongated, longer than wide in dorsal view, with numerous setae. Total length of specimen is 2.00 mm.

11. Specimen 3223 (BUB 3063), preserved in Cretaceous Kachin amber, Myanmar. Many details of the specimen are not well accessible in dorsal (Figure 11A,B) and ventral view (Figure 11C), due to a reflective film covering the specimen. Head capsule roughly square-shaped in dorsal view, only slightly longer than wide; stylets longer than head capsule (Figure 11D). Stemmata on slight elevations on each side of head capsule (Figure 11D). Antennae small, very slender, far lateral, slightly anterior to stemmata. Each stylet has two major teeth and few smaller teeth or robust setae (Figure 11A–C). No palp apparent. Pronotum cap-like. No apparent protrusions on trunk segments. Thorax segments ventrally with locomotory appendages. Legs distally with three prominent elements (femur, tibia, tarsus), with the tibia and tarsus well separated (Figure 11E). Distally tarsus with pair of claws, no empodium apparent. Trunk end is elongated, longer than wide in dorsal view, with numerous setae. Total length of specimen is 4.97 mm.

12. Specimen 3224 (BUB 3724), preserved in Cretaceous Kachin amber, Myanmar. Specimen is complete and is well accessible in ventral (Figure 12A) and dorsal view (Figure 12B,C), yet partly concealed by debris, apparently attached to the body forming camouflaging cloak. Head capsule roughly square-shaped in dorsal view, only slightly wider than long (Figure 12D); stylets longer than head capsule (Figure 12D). Stemmata on prominent protrusions on each side of head capsule (Figure 12D). Antennae small, very slender, far lateral, slightly anterior to stemmata. Each stylet has two major teeth and few smaller teeth or robust setae (Figure 12A–D). Labial palp is short, robust. Pronotum cap-like. No apparent protrusions on trunk segments. Thorax segments ventrally with locomotory appendages. Legs distally with three prominent elements (femur, tibia, tarsus), with the tibia and tarsus well separated (Figure 12E). Distal tarsus with a pair of claws, no empodium apparent. Trunk end is elongated, longer than wide in dorsal view, with numerous setae. Total length of specimen is 3.68 mm.

13. Specimen 3225 (CJW F 3199), preserved in Cretaceous Kachin amber, Myanmar. Specimen is accessible in oblique dorsal (Figure 13A,B) and oblique ventral view (Figure 13C). Head capsule roughly square-shaped in dorsal view, only slightly longer than wide; stylets about the same length as head capsule (Figure 13A–C). Stemmata on prominent protrusions on each side of head capsule, but obscured on one side (Figure 13D). Antennae small, very slender, far lateral, slightly anterior to stemmata. Each stylet has two major teeth and few smaller teeth or robust setae (Figure 13A,B). No palps apparent. Pronotum cap-like. Thorax segments ventrally with locomotory appendages. Legs distally with three prominent elements (femur, tibia, tarsus), with the tibia and tarsus well separated (Figure 13E). Total length of specimen is 2.68 mm.

14. Specimen 3226 (CJW F 3421), preserved in Cretaceous Kachin amber. Specimen is not well accessible, strongly folded, body visible in oblique lateral view (Figure 13F,G), head also in ventral view (Figure 13H,I). Body largely concealed by debris, apparently attached to the body forming a camouflaging cloak. Head capsule roughly square-shaped in dorsal view, only slightly longer than wide; stylets longer than head capsule (Figure 13H,I). Thorax segments ventrally with locomotory appendages. Legs distally with three prominent elements (femur, tibia, tarsus), with the tibia and tarsus well separated (Figure 13F,G). Total length of specimen is 2.72 mm.

15. Specimen 3227 (PED 0083a), preserved in Cretaceous Kachin amber, Myanmar. Specimen is well accessible in ventral view (Figure 14A,B), trunk partly folded. Head capsule roughly square-shaped in ventral view; stylets longer than head capsule (Figure 14A,B). Stemmata on prominent protrusions on each side of head capsule (Figure 14A,B). Anterior rim with simple setae. Antennae small, very slender, far lateral, slightly anterior to stemmata. Each stylet has two major teeth and few smaller teeth or robust setae (Figure 14A,B). No palps apparent. Mesothorax and metathorax with prominent dorso-lateral protrusions bearing numerous setae (Figure 14A,B). Thorax segments ventrally with locomotory appendages. Legs distally with three prominent elements (femur, tibia, tarsus), with the tibia and tarsus well separated (Figure 14C,D). Distal tarsus with a pair of claws, no empodium apparent (Figure 14C,D). Anterior abdomen segments also with protrusions, but smaller ones. Trunk end is elongated, longer than wide in dorsal view (folded forwards, therefore seen from dorsally), with numerous setae. Total length of specimen is 3.01 mm.

16. Specimen 3228 (PED 0083b), preserved in Cretaceous Kachin amber, Myanmar, same piece as previous specimen. Specimen is well accessible in mostly ventral view (Figure 15A,B), but partly twisted and folded, hence the posterior trunk is seen in the dorsal view. Head capsule roughly square-shaped in dorsal view, only slightly wider than long; stylets longer than head capsule (Figure 15A,B). Anterior rim with simple setae. Stemmata on prominent protrusions on each side of head capsule (Figure 15D). Antennae small, very slender, far lateral, slightly anterior to stemmata. Each stylet has two major teeth and few smaller teeth or robust setae (Figure 15C). Labial palp is short, robust. Thorax segments ventrally with locomotory appendages. Legs distally with three prominent elements (femur, tibia, tarsus), with the tibia and tarsus well separated (Figure 15E). Distal tarsus with a pair of claws, no empodium apparent. Anterior abdomen segments with protrusions. Trunk end is elongate, longer than wide in dorsal view, with numerous setae. Total length of specimen is 3.60 mm.

17. Specimen 3229 (PED 0083c), preserved in Cretaceous Kachin amber, Myanmar, same piece as two previous specimens. Specimen is well accessible in slightly oblique ventral view (Figure 16A,B), not well accessible in dorsal view, partially concealed by other individuals (Figure 16C). Head capsule roughly square-shaped in dorsal view, only slightly wider than long; stylets longer than head capsule (Figure 16A,B). Anterior rim with simple setae. Stemmata on prominent protrusions on each side of head capsule (Figure 16D). Antennae small, very slender, far lateral, slightly anterior to stemmata. Each stylet has two major teeth and few smaller teeth or robust setae (Figure 16A,B). Labial palp is short, robust. Thorax segments ventrally with locomotory appendages. Legs distally with three prominent elements (femur, tibia, tarsus), with the tibia and tarsus well separated (Figure 16E). Distal tarsus with a pair of claws, no empodium apparent. Anterior abdomen segments with protrusions. Trunk end is elongated, longer than wide in dorsal view, with numerous setae. Total length of specimen is 3.48 mm.

18. Specimen 3230 (PED 0083d), preserved in Cretaceous Kachin amber, Myanmar, same piece as three previous specimens. Specimen is well accessible in slightly oblique dorsal view (Figure 17A,B), and is less well accessible in ventral view (Figure 17C), partly obscured by other individuals. Head capsule roughly square-shaped in dorsal view; stylets longer than head capsule (Figure 17A,B). Anterior rim with simple setae. Stemmata on elevations on each side of head capsule (Figure 17C). Antennae small, very slender, far lateral, slightly anterior to stemmata. Each stylet has two major teeth and few smaller teeth or robust setae (Figure 17D). Labial palp is short, robust. Pronotum is cap-like. Mesothorax and metathorax with very prominent dorso-lateral protrusions bearing numerous setae (Figure 17A,B). Thorax segments ventrally with locomotory appendages. Legs distally with three prominent elements (femur, tibia, tarsus), with the tibia and tarsus well separated (Figure 17E). Distal tarsus with a pair of claws, no empodium apparent. Anterior abdomen segments also with protrusions, but smaller ones. Trunk end is elongated, longer than wide in dorsal view, with numerous setae. Total length of specimen is 3.62 mm.

19. Specimen 3231 (PED 0083e), preserved in Cretaceous Kachin amber, Myanmar, same piece as four previous specimens. Largely concealed by other individuals, mostly the head is accessible in the dorsal (Figure 18A,B) and ventral view (Figure 18C). Head capsule roughly square-shaped in dorsal view, slightly wider than long; stylets longer than head capsule (Figure 18A–C). Stemmata on slight elevations on each side of head capsule (Figure 18A–C). Antennae small, very slender, far lateral, slightly anterior to stemmata. Each stylet bears two major teeth and few smaller teeth or robust setae (Figure 18A–C). Preserved part of specimen is 1.96 mm.

20. An additional partial specimen (PED 0083f) is preserved within the same amber piece as the five previous specimens. Yet, the specimen (Figure 18D,E) is too incomplete to further consider it here.

21. Specimen 3232 (PED 0087), preserved in Cretaceous Kachin amber, Myanmar. Specimen is well accessible in ventral (Figure 19A) and dorsal view (Figure 19B,C). Head capsule roughly square-shaped in dorsal view; stylets longer than head capsule (Figure 19A–C). Stemmata on prominent protrusions on each side of head capsule (Figure 19A–C). Antennae small, very slender, far lateral, slightly anterior to stemmata. Each stylet has two major teeth and few smaller teeth or robust setae (Figure 19E). Labial palp is short, robust. Thorax segments ventrally with locomotory appendages. Legs distally with three prominent elements (femur, tibia, tarsus), with the tibia and tarsus well separated (Figure 19D). Distal tarsus with a pair of claws, no empodium apparent. Anterior abdomen segments with slight protrusions. Trunk end is elongated, longer than wide in dorsal view, with numerous setae. Total length of specimen is 3.76 mm.

22. Specimen 3233 (PED 0249), preserved in Cretaceous Kachin amber, Myanmar. Head of specimen is well accessible in dorsal (Figure 20A,B) and ventral view (Figure 20C). Trunk partly concealed by debris, apparently attached to the body forming a camouflaging cloak. Posterior end of trunk seems not to be preserved inside the amber piece. Head capsule roughly square-shaped in dorsal view; stylets longer than head capsule (Figure 20A–C). Stemmata on protrusions on each side of head capsule (Figure 20E). Antennae small, very slender, far lateral, slightly anterior to stemmata. Each stylet has two major teeth and few smaller teeth or robust setae (Figure 20A–C). Labial palp is short, robust. Thorax segments ventrally with locomotory appendages. Legs distally with three prominent elements (femur, tibia, tarsus), with the tibia and tarsus well separated (Figure 20D). Distal tarsus with a pair of claws, no empodium apparent. Abdomen segments not accessible. Preserved part of specimen is 4.47 mm.

23. Specimen 3234 (PED 0272), preserved in Cretaceous Kachin amber, Myanmar. Specimen is well accessible in dorsal view (Figure 21A,B), ventrally largely concealed by dirt particles (Figure 21C). Head capsule roughly square-shaped in dorsal view, only slightly wider than long; stylets longer than head capsule (Figure 21A,B). Anterior rim with deeply forked spine, one on each side of midline (Figure 21D). Stemmata on prominent protrusions on each side of head capsule (Figure 21D). Antennae small, very slender, far lateral, slightly anterior to stemmata. Each stylet has two major teeth and few smaller teeth or robust setae (Figure 21A–C). No palps apparent. Pronotum cap-like. Mesothorax and metathorax with very prominent dorso-lateral protrusions bearing numerous setae (Figure 21E). Thorax segments ventrally with locomotory appendages. Legs distally with three prominent elements (femur, tibia, tarsus), with the tibia and tarsus well separated. Anterior abdomen segments also with protrusions, but smaller ones. Trunk end is elongated, longer than wide in dorsal view, with numerous setae. Total length of specimen is 2.23 mm.

24. Specimen 3235 (PED 0282) is preserved in Cretaceous Kachin amber, Myanmar. Specimen is well accessible in dorsal (Figure 22A,B) and ventral view (Figure 22C), with the further posterior part of trunk slightly concealed by debris, apparently attached to the body forming a camouflaging cloak. Head capsule roughly square-shaped in dorsal view, slightly wider than long; stylets longer than head capsule (Figure 22A–C). Stemmata on protrusions on each side of head capsule (Figure 22D). Antennae and palps not apparent, only very proximal part of antennae visible. Each stylet has two major teeth and few smaller teeth or robust setae (Figure 22D). Thorax segments ventrally with locomotory appendages. Legs distally with three prominent elements (femur, tibia, tarsus), with the tibia and tarsus well separated (Figure 22E). Distal tarsus with a pair of claws, no empodium apparent. Anterior abdomen segments also with protrusions, but smaller ones. Total length of specimen is 5.54 mm.

25. Specimen 3236 (PED 0318), preserved in Cretaceous Kachin amber, Myanmar. Specimen is accessible in ventral (Figure 23A,B) and dorsal view (Figure 23C). Head capsule roughly square-shaped in dorsal view, slightly longer than wide; stylets slightly longer than head capsule (Figure 23E). Stemmata on elevations on each side of head capsule (Figure 23E). Antennae small, very slender, far lateral, slightly anterior to stemmata (Figure 23F). Each stylet has two major teeth and few smaller teeth or robust setae (Figure 23E). No palps apparent. Thorax segments ventrally with locomotory appendages. Legs distally with three prominent elements (femur, tibia, tarsus), with the tibia and tarsus well separated (Figure 23D). Distal tarsus with a pair of claws, no empodium apparent. Anterior abdomen segments also with protrusions, but smaller ones. Trunk end is elongated, longer than wide in dorsal view, with numerous setae. Total length of specimen is 7.35 mm.

26. Specimen 3237 (PED 0319), preserved in Cretaceous Kachin amber, Myanmar. Specimen is well accessible in dorsal view (Figure 24A,B), ventrally partly concealed by irregularities in the amber (Figure 24C). Head capsule roughly square-shaped in dorsal view, only slightly wider than long; stylets longer than head capsule (Figure 24A–C). Anterior rim with simple setae. Stemmata on prominent protrusions on each side of head capsule (Figure 24D). Antennae small, very slender, far lateral, slightly anterior to stemmata. Each stylet has two major teeth and few smaller teeth or robust setae (Figure 24D). Labial palp short, robust. Metathorax with very prominent dorso-lateral protrusions bearing numerous setae (Figure 24A,B). Thorax segments ventrally with locomotory appendages. Legs distally with three prominent elements (femur, tibia, tarsus), with the tibia and tarsus well separated (Figure 24E). Distal tarsus with a pair of claws, no empodium apparent. Abdomen not preserved. Total length of specimen is 3.78 mm.

27. Specimen 3238 (PED 0320), preserved in Cretaceous Kachin amber, Myanmar. Specimen is partly concealed by irregularities of the amber in ventral view (Figure 25A), dorsally better accessible (Figure 25B,C). Head capsule roughly square-shaped in dorsal view, only slightly wider than long; stylets longer than head capsule (Figure 25A–C). Stemmata on prominent protrusions on each side of head capsule (Figure 25E). Antennae small, very slender, far lateral, slightly anterior to stemmata. Each stylet has two major teeth and few smaller teeth or robust setae (Figure 25D). Pronotum cap-like. Mesothorax and metathorax with very prominent dorso-lateral protrusions bearing numerous setae (Figure 25B,C,F). Thorax segments ventrally with locomotory appendages. Legs distally with three prominent elements (femur, tibia, tarsus), with the tibia and tarsus well separated (Figure 25G). Distally tarsus with pair of claws, no empodium apparent. Trunk end is elongated rounded, with numerous setae. Total length of specimen is 4.00 mm.

28. Specimen 3239 (PED 0378) is preserved in Cretaceous Kachin amber, Myanmar. Specimen is accessible in dorsal view (Figure 26A,B), ventrally largely concealed (Figure 26C), trunk only preserved as outline. Head capsule roughly square-shaped in dorsal view, only slightly wider than long; stylets longer than head capsule (Figure 26D). Stemmata on prominent protrusions on each side of head capsule (Figure 26D). Antennae small, very slender, far lateral, slightly anterior to stemmata. Each stylet has two major teeth and few smaller teeth or robust setae (Figure 26D). No palps apparent. Thorax is largely concealed by debris, apparently attached to the body forming a camouflaging cloak. Thorax segments ventrally with locomotory appendages. Legs distally with three prominent elements (femur, tibia, tarsus), with he tibia and tarsus well separated (Figure 26E). Distal tarsus with a pair of claws, no empodium apparent. Further details of trunk not available. Total length of specimen is 4.30 mm.

29. Specimen 3240 (PED 0520), preserved in Cretaceous Kachin amber, Myanmar. Specimen is well accessible in ventral (Figure 27A) and dorsal view (Figure 27B,C). Head capsule roughly square-shaped in dorsal view, only slightly wider than long; stylets longer than head capsule (Figure 27A–C). Anterior rim with simple setae. Stemmata on prominent protrusions on each side of head capsule (Figure 27D). Antennae small, very slender, far lateral, slightly anterior to stemmata. Each stylet has two major teeth and few smaller teeth or robust setae (Figure 27A–C). No palps apparent. Pronotum cap-like. Mesothorax and metathorax with very prominent dorso-lateral protrusions bearing numerous setae (Figure 27F). Thorax segments ventrally with locomotory appendages. Distal tarsus with pair of claws, no empodium apparent (Figure 27E). Anterior abdomen segments also with protrusions, but smaller ones. Trunk end not apparent due to air bubble. Total length of specimen is 5.20 mm.

30. Specimen 3241 (PED 0563), preserved in Cretaceous Kachin amber, Myanmar. Specimen is accessible in dorsal view (Figure 28A,B); many details not accessible due to numerous small dirt particles; posterior end of trunk not preserved. Head capsule roughly square-shaped in dorsal view; stylets longer than head capsule (Figure 28A,B). Stemmata on elevations on each side of head capsule (Figure 28C). Antennae small, very slender, far lateral, slightly anterior to stemmata. Each stylet has two major teeth and few smaller teeth or robust setae (Figure 28C). No palps apparent. Thorax segments ventrally with locomotory appendages. Legs distally with three prominent elements (femur, tibia, tarsus), with the tibia and tarsus well separated (Figure 28D). Preserved part of specimen is 3.33 mm.

31. Specimen 3242 (PED 0575), preserved in Cretaceous Kachin amber, Myanmar. Specimen is not well accessible, neither in the dorsal (Figure 29A,B) nor ventral view (Figure 29C). Head capsule roughly square-shaped in dorsal view; stylets about as long as head capsule (Figure 29A–C). Stemmata on elevations on each side of head capsule (Figure 29D). Antennae small, very slender, far lateral, slightly anterior to stemmata. Each stylet has two major teeth (Figure 29A–C). No palps apparent. Thorax segments ventrally with locomotory appendages. Legs distally with three prominent elements (femur, tibia, tarsus), with the tibia and tarsus well separated (Figure 29E). Distal tarsus with a pair of claws, no empodium apparent. Details of posterior trunk not accessible. Preserved part of specimen is 4.2 mm.

32. Specimen 3243 (PED 0583), preserved in Cretaceous Kachin amber, Myanmar. Specimen is accessible in ventral (Figure 30A,B) and dorsal view (Figure 30C), yet dirt and other impurities conceal many details. Head capsule roughly square-shaped in dorsal view, only slightly longer than wide; stylets longer than head capsule (Figure 30A–C). Stemmata on prominent protrusions on each side of head capsule (Figure 30D). Antennae small, very slender, far lateral, slightly anterior to stemmata. Each stylet has two major teeth and few smaller teeth or robust setae (Figure 30A–C). No palps apparent. Thorax segments ventrally with locomotory appendages. Legs distally with two prominent elements (tibia, tarsus), which are well separated (Figure 30E). Distal tarsus with a pair of claws, no empodium apparent. No details of abdomen segments accessible. Trunk end is elongated, longer than wide in ventral view, with numerous setae. Total length of specimen is 8.20 mm.

33. Specimen 3244 (PED 0684), preserved in Cretaceous Kachin amber, Myanmar. Specimen is accessible in dorsal view (Figure 31A,B), largely concealed in ventral view (Figure 31C), posterior end incomplete, damaged. Head capsule roughly square-shaped in dorsal view, only slightly longer than wide; stylets about as long as head capsule (Figure 31A–C). Stemmata on elevations on each side of head capsule (Figure 31D). Antennae small, very slender, far lateral, slightly anterior to stemmata. Each stylet has two major teeth (Figure 31A–C). No palps apparent. Mesothorax and metathorax with prominent dorso-lateral protrusions bearing numerous setae (Figure 31A,B). Thorax segments ventrally with locomotory appendages. Legs distally with three prominent elements (femur, tibia, tarsus), with the tibia and tarsus well separated (Figure 31E). Distal tarsus with a pair of claws, no empodium apparent. Details of abdomen segments not well accessible. Preserved part of specimen is 8.06 mm.

34. Specimen 3245 (PED 0944), preserved in Cretaceous Kachin amber, Myanmar. Specimen is well accessible in the dorsal (Figure 32A,B) and ventral view (Figure 32C). Head capsule roughly square-shaped in dorsal view, but wider than long; stylets longer than head capsule (Figure 32D). Anterior rim with deeply forked spine, one on each side of midline (Figure 32D). Stemmata on prominent protrusions on each side of head capsule (Figure 32D). Antennae small, very slender, far lateral, slightly anterior to stemmata. Each stylet has two major teeth and few smaller teeth or robust setae (Figure 32D). Labial palp short, robust. Pronotum cap-like. Mesothorax and metathorax with very prominent dorso-lateral protrusions bearing numerous setae (Figure 32A,B). Thorax segments ventrally with locomotory appendages. Legs distally with three prominent elements (femur, tibia, tarsus), with the tibia and tarsus well separated (Figure 32E–G), also in leg 3 (Figure 32E). Distal tarsus with a pair of claws, no empodium apparent. Anterior abdomen segments also with protrusions, but smaller ones. Trunk end is elongated, longer than wide in dorsal view, with numerous setae. Total length of specimen is 2.07 mm.

35. Specimen 3246 (PED 0975), preserved in Cretaceous Kachin amber, Myanmar. Specimen is accessible in dorsal (Figure 33A), ventral (Figure 33B,C) and lateral view (Figure 33D). Head capsule roughly square-shaped in dorsal view, but slightly wider than long; stylets longer than head capsule (Figure 33A–C). Stemmata on prominent protrusions on each side of head capsule (Figure 33E). Antennae small, very slender, far lateral, slightly anterior to stemmata. Each stylet has two major teeth and few smaller teeth or robust setae (Figure 33A,B). No palps apparent. Mesothorax and metathorax with very prominent dorso-lateral protrusions bearing numerous setae (Figure 33A,B). Thorax segments ventrally with locomotory appendages. Legs distally with three prominent elements (femur, tibia, tarsus), with the tibia and tarsus well separated (Figure 33F). Distal tarsus with a pair of claws, no empodium apparent. Abdomen segments not well accessible. Total length of specimen is 2.65 mm.

36. Specimen 3247 (PED 1047), preserved in Cretaceous Kachin amber, Myanmar. Specimen is accessible in dorsal view (Figure 34A,B). Head capsule roughly square-shaped in dorsal view, only slightly wider than long; stylets longer than head capsule (Figure 34C). Anterior rim with deeply forked spine, one on each side of midline (Figure 34C). Stemmata on prominent protrusions on each side of head capsule (Figure 34C). Antennae are small, very slender, far lateral, slightly anterior to stemmata. Each stylet has two major teeth and few smaller teeth or robust setae (Figure 34C). Labial palp short, robust. Pronotum cap-like. Mesothorax and metathorax with very prominent dorso-lateral protrusions bearing numerous setae (Figure 34A,B). Thorax segments ventrally with locomotory appendages. Legs distally with three prominent elements (femur, tibia, tarsus), with the tibia and tarsus well separated (Figure 34D). Anterior abdomen segments also with protrusions, but smaller ones. Trunk end is elongated, longer than wide in dorsal view, with numerous setae. Total length of specimen is 5.35 mm.

37. Specimen 3248 (PED 1206), preserved in Cretaceous Kachin amber, Myanmar. Only the head is preserved. Specimen is well accessible in dorsal and ventral view (Figure 35A–C). Head capsule roughly square-shaped in dorsal view; stylets longer than head capsule (Figure 35A–C). Stemmata on prominent protrusions on each side of head capsule (Figure 35D,E). Each stylet has two major teeth and numerous smaller teeth or robust setae (Figure 35A–C). Anterior rim medially with deeply forked spine, one on each side of midline (Figure 35A–C). Antennae are small, very slender, far lateral, slightly anterior to stemmata. No palps apparent. Preserved part of specimen is 2.04 mm.

38. Specimen 3249 (Weiterschan BuB 23), preserved in Cretaceous Kachin amber, Myanmar. Specimen is well accessible in ventral view (Figure 36A,B), largely concealed in dorsal view (Figure 36C). Head capsule roughly square-shaped in dorsal view, slightly wider than long; stylets longer than head capsule (Figure 36A–C). Stemmata on prominent protrusions on each side of head capsule (Figure 36D). Antennae small, very slender, far lateral, slightly anterior to stemmata. Each stylet has two major teeth and few smaller teeth or robust setae (Figure 36A–C). No palps apparent. Mesothorax and metathorax with very prominent dorso-lateral protrusions bearing numerous setae (Figure 36A,B). Thorax segments ventrally with locomotory appendages. Legs distally with three prominent elements (femur, tibia, tarsus), with the tibia and tarsus well separated (Figure 36E). Anterior abdomen segments also with protrusions, but smaller ones. Trunk end about as long as wide in dorsal view, with numerous setae. Total length of specimen is 3.68 mm.

### 3.2. Shape Analysis

The analysis resulted in a total of three effective principal components (Supplementary File S3). These together explain 93.8% of the overall variation (Supplementary Files S3–S5). 

PC1 explains 72.4% of the overall variation. It mostly describes the general form of the head capsule and stylets. Positive values indicate a concave posterior rim of the head capsule with mandibles mostly aligned with the head capsule. Negative values indicate a convex posterior rim of the head capsule with mandibles curved outwards and possibly with teeth near the end of the stylets (Supplementary File S4).

PC2 explains 17.9% of the overall variation. It mostly describes the relative length of the head capsule and stylets. Positive values indicate relatively shorter stylets and a compact head capsule. Negative values indicate relatively longer stylets (Supplementary File S4).

PC3 explains 3.4% of the overall variation. It mostly describes the relative thickness of the stylets. A positive value indicates a thicker mandible. A negative value indicates a thinner mandible (Supplementary File S4). 

### 3.3. Statistical Differences in the Morphospace of Different Time Groups

The average displacement of individuals and the sum of variance was compared between the two major groups, fossil vs. extant. The randomized sampling tests show a very significant (*p* < 0.01) corrected difference in the size of the groups in the morphospace. The PERMANOVA analysis shows a very significant (*p* < 0.01) difference in position of the groups in the morphospace.

## 4. Discussion

### 4.1. Identity of the Specimens Reported Here

The single new specimen from the Eocene reported here is fairly incomplete, yet the preserved morphology clearly indicates that this specimen is a representative of Ascalaphidae (or Ascalaphinae). As many other details are not available (antennae, trunk structures), we can not further narrow down the relationship (or taxonomic identity) of this specimen.

Badano et al. [50] formally described three species based on fossil larvae from Myanmar, with two teeth on each mandible (similar to most of the larvae reported here): *Diodontognathus papillatus*, *Mesoptynx unguiculatus*, and *Adelpholeon lithophorus*. Similar-appearing larvae seem to have generally been interpreted as larvae of Ascalaphidae (e.g., [47], Figure 6 p. 6), but were identified in Badano et al. [50] as being representatives of the larger group, including all representatives traditionally considered Ascalaphidae and Myrmeleontidae. All these three fossil species described in Badano et al. [50] differ from extant representatives of this larger group by having the tibia and tarsus well separated (“not fused”) on trunk appendage three. This condition is also the case in the specimens reported here from the Myanmar amber in which this detail is available.

It is possible that all the larvae from Myanmar reported here are representatives of one of the three species described by Badano et al. [50]. Unfortunately, the differences of the three species are linked to very specific characteristics (e.g., of the spiracles), which are not available in most of the specimens at hand. It is also possible that some of the larvae reported here represent different instars of the already described species, but differ in certain aspects from them due to the different ontogenetic state. It can currently not be excluded that also new species are represented in the material reported here. The morphology of the new larvae clearly identifies them as being closely related to the three species, with owllion-type larvae, and may be (in part?) conspecific to them, but this aspect cannot be further evaluated.

The single specimen from French amber has been interpreted as a representative of Ascalaphidae [47]. Due to the incomplete preservation, it remains difficult to further evaluate this interpretation. Yet, given the uncertainty of the other larvae it seems more careful to interpret this specimen also as a representative of the larger group, including the extant forms generally interpreted as Myrmeleontidae and Ascalaphidae as well as the three species described by Badano et al. [50]. 

The taxonomic uncertainty of the larvae might be unsatisfying. Still at least the coarse relationship of these larvae could be identified, allowing a basis for a morphological comparison.

### 4.2. Sub-Sample Sizes

As in many earlier analyses [78,79,81,82,83] (but not all, see [75]), the sub-sample size for the extant fauna is the largest. The sub-sample size of the Cretaceous larvae is the largest of the fossil faunas. It is an interesting detail that there are significantly more larvae in Miocene ambers than in Eocene ones. This ratio is quite different for other lacewing groups (e.g., [62]), as the Eocene amber, especially Baltic amber, is known for a much larger number of amber pieces than Miocene amber. Despite the larger sample size of the Miocene specimens, the sub-sample size of the Miocene and Eocene is still quite small, and a most reliable comparison is to be expected from the Cretaceous versus the extant fauna. However, the other time slices will also be considered for a broader comparison. For other aspects concerning the differences in sample sizes, data availability, or data quality, see for example [75].

### 4.3. Morphospace Occupation of the Extant Larvae

**Figure 37:**
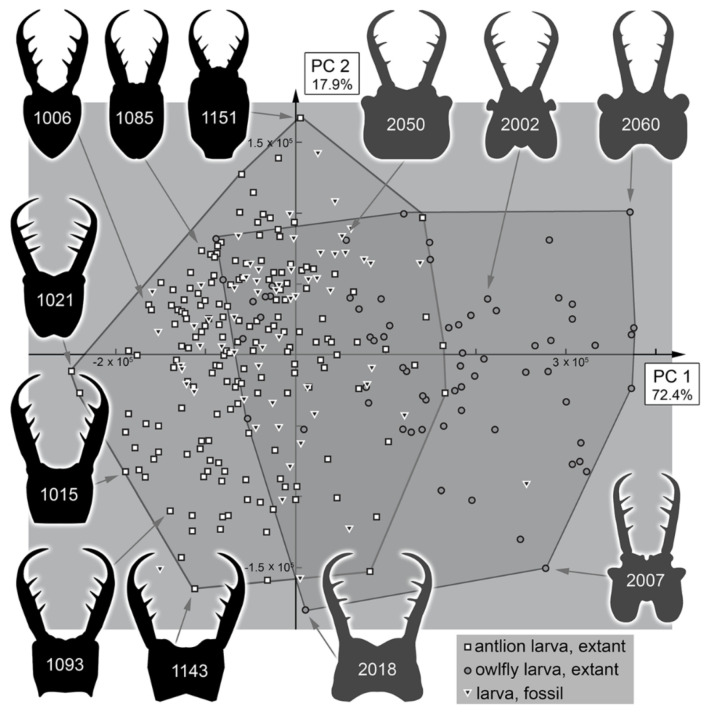
Scatterplot of PC2 vs. PC1 values of head shapes of all specimens. Black heads = antlion larvae; grey heads = owlfly larvae.

**Figure 38:**
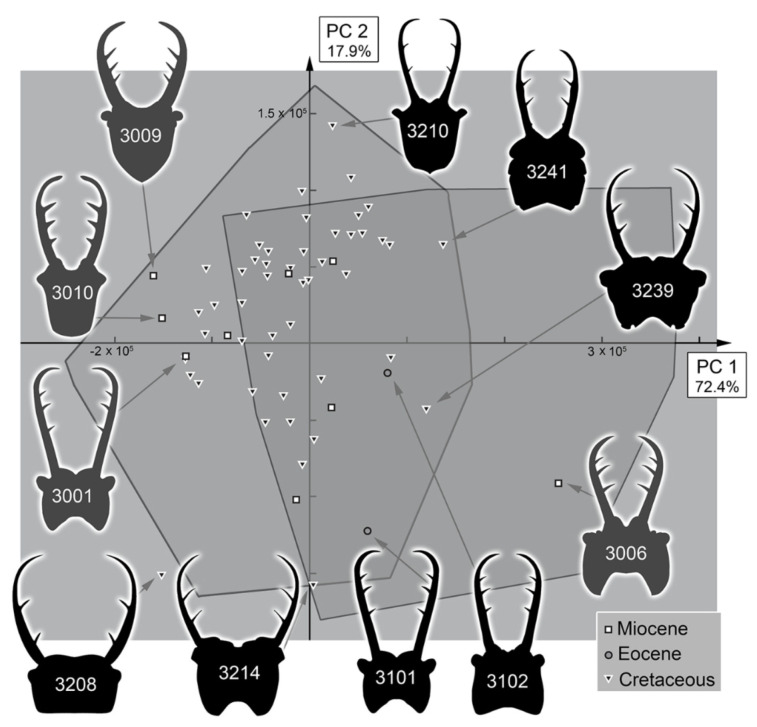
Scatterplot of PC2 vs. PC1 values of head shapes of fossil specimens. Extant specimens only shown as occupied area to highlight position of fossil specimens. Black heads = Cretaceous and Eocene larvae; grey heads = Miocene larvae.

**Figure 39:**
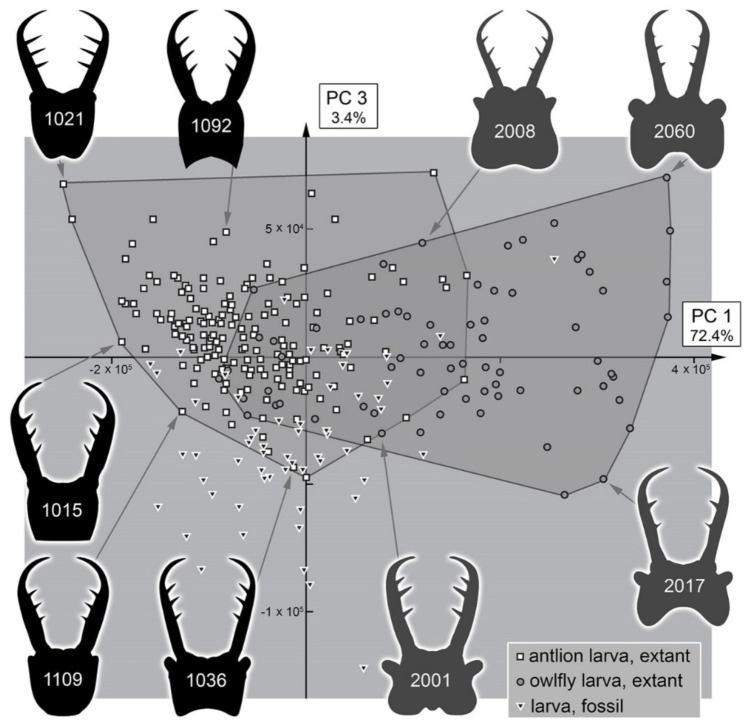
Scatterplot of PC3 vs. PC1 values of head shapes of all specimens. Black heads = antlion larvae; grey heads = owlfly larvae.

**Figure 40:**
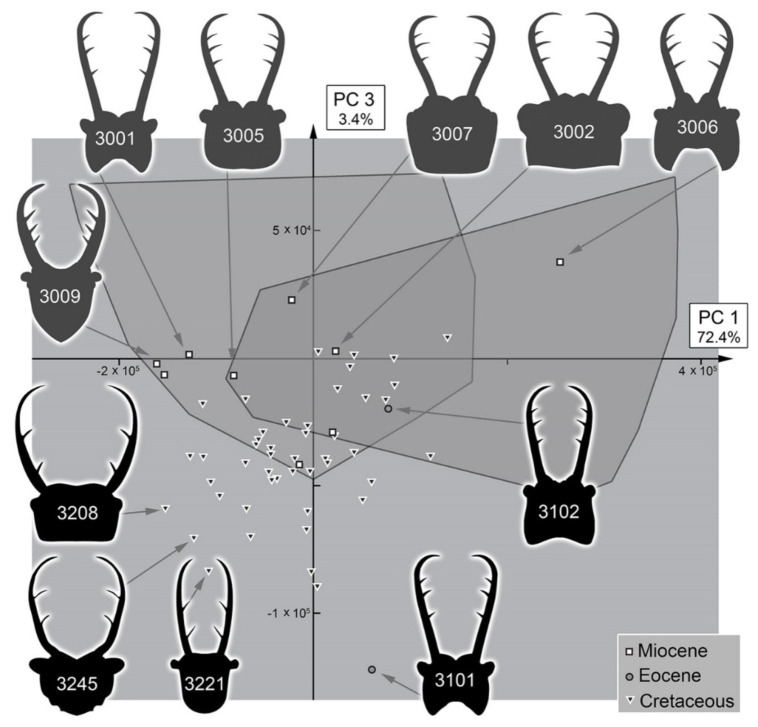
Scatterplot of PC3 vs. PC1 values of head shapes of fossil specimens. Extant specimens only shown as an occupied area to highlight the position of fossil specimens. Black heads = Cretaceous and Eocene larvae; grey heads = Miocene larvae.

When comparing the shapes of the heads of the extant larvae, it becomes apparent that Myrmeleontidae-type larvae and Ascalaphidae-type larvae largely overlap (Figure 37, Figure 38, Figure 39 and Figure 40). Given their overall complicated but close relationship, this overlap is not surprising. There are also some areas in which only one of the two types is represented (Figure 37 and Figure 39). In the right area of the morphospace (positive PC1 values), the heads with strongly concave posterior edges are plotted, which exclusively occur in Ascalaphidae-type larvae; in the left area, either relatively shorter or posteriorly convex heads are ploted, which exclusively occur in some Myrmeleontidae-type larvae. 

### 4.4. Morphospace Occupation of the Fossil Larvae

All the larvae from the Miocene are plotted inside the area occupied by extant larvae (Figure 38 and Figure 40). As all these specimens seem to be representatives of extant lineages, this finding is not surprising. Additionally, there are two larvae from the Eocene plotted within the range of the extant forms when considering PC1 and PC2 (Figure 38). However, one of the two larvae is plotted outside the range of extant larvae when considering PC3 (Figure 40). It appears that the fossil differs from the extant forms in the relatively longer stylets. 

The larvae from the Cretaceous are also plotted inside the area of extant forms considering PC1 and PC2, yet the overall area is significantly smaller (Figure 38). Quite a number of specimens are plotted outside the area occupied by extant forms when considering PC3, similar to the one larva from the Eocene, yet the Eocene larva in fact is even plotted further outside than the Cretaceous ones (Figure 40). 

It is interesting to note that, despite the fact that most Cretaceous larvae have been originally addressed as larvae of Ascalaphidae, not a single one plots in the area exclusively occupied by extant Ascalaphidae-type larvae for PC2 vs. PC1 (Figure 38), and only a single one plots there for PC3 vs. PC1 (Figure 40). Even more so, some of the fossil specimens are even plotted outside the area of extant Ascalaphidae-type larvae in the area of extant Myrmeleontidae-type larvae (Figure 38 and Figure 40). 

### 4.5. Diversity Changes through Time in Owllion Larvae

As apparent by the comparison of the morphospace occupation, the extant fauna has the highest morphological diversity. This difference in diversity indicates a diversification of larval forms in the extant lineages traditionally addressed as being Ascalaphidae and Myrmeleontidae, which may not be surprising concerning the Myrmeleontidae-type larvae, yet it is partly surprising concerning the Ascalaphidae-type larvae. As pointed out, many of the Cretaceous larvae have generally been interpreted as Ascalaphidae-type larvae. Even some reconstructed behavioral aspects of such larvae are remarkably comparable to extant Ascalaphidae-type larvae [86]. Still, in quantitative morphological aspects, the head and mandible of the Cretaceous fossils apparently differ from extant Ascalaphidae-type larvae. 

Despite the fact that, in major aspects, the extant larvae show a higher morphological diversity than the Cretaceous fossils, when considering PC3, the fossil larvae have values that were not found among the extant representatives. Therefore, there are still certain morphologies that have disappeared and are no longer present in the extant fauna. Hence, while there is a net increase of the morphological diversity of owllion larvae, there are also certain morphologies that have been lost. Interestingly, there is an Eocene larva that also represents such a lost type of morphology as a kind of “long-term survivor”. 

### 4.6. Diversity Changes through Time in Comparison to Other Myrmeleontiformian Lacewings

In closely related lineages to owlflies, such as silky lacewings (Psychopsidae) and split-footed lacewings (Nymphidae), the diversity has decreased over time [75,82]. In other lineages, thread-winged lacewings (Crocinae) and spoon-winged lacewings (Nemopterinae), the case is less easy to evaluate due to a lower data availability [78,79].

The diversification of Myrmeleontidae-type larvae may have been coupled to the evolution of a new strategy, namely digging [50], as well as, in some ingroups, pit-building. For Ascalaphidae-type larvae, the diversification may have been related to the decrease of diversity in Nymphidae. The larvae of Nymphidae, especially those of Myiodactylinae, possess quite similar morphologies especially concerning their trunk shapes and processes, both coupled to attaching a camouflaging cloak (e.g., [47]). Hence, it seems reasonable that some ecological functions fulfilled by larvae of Nymphidae in the Cretaceous are today fulfilled by Ascalaphidae-type larvae, not indicating that the latter drove the former out. Probably also further factors played an important role here, for example, the flight abilities or other ecological aspects of the adults. 

Despite the few owllion larvae in the Eocene, there is similarity to silky lacewing larvae: there are larvae in the Eocene that differ in morphology from their extant counterparts. This observation further emphasizes that the Eocene fauna is not identical to the extant one, but also that the faunal change for Myrmeleontiformia was not a single event at the end of the Cretaceous, but a more continuous process.

## Figures and Tables

**Figure 12:**
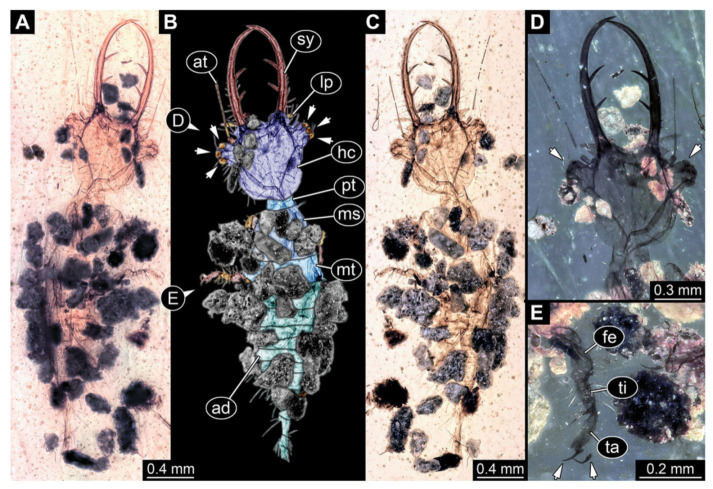
Fossil owllion larva, specimen 3224 (BUB 3724), Cretaceous Kachin amber, Myanmar. (**A**) Overview, ventral. (**B**) Color-marked version of (**C**); arrows point to stemmata. (**C**) Overview, dorsal. (**D**) Close-up on head capsule with fields of stemmata (arrows). (**E**) Close-up on locomotory appendage with claws (arrows). Abbreviations: ad = abdomen; at = antenna; fe = femur; hc = head capsule; lp = labial palp; ms = mesothorax; mt = metathorax; pt = prothorax; sy = stylet; ta = tarsus; ti = tibia.

**Figure 13:**
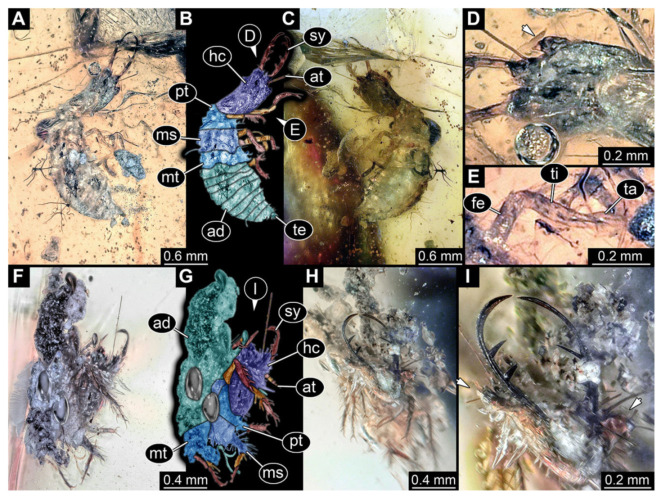
Fossil owllion larvae, Cretaceous Kachin amber, Myanmar. (**A**–**E**) Specimen 3225 (CJW F 3199). (**A**) Overview, oblique dorsal. (**B**) Color-marked version of (**A**). (**C**) Overview, oblique ventral. (**D**) Close-up on head capsule with field of stemmata (arrow). I Close-up on locomotory appendage. (**F**–**I**) Specimen 3226 (CJW F 3421). (**F**) Overview, antero-lateral. (**G**) Color-marked version of (**F**). (**H**) Overview, anterior. (**I**) Close-up on head capsule, with fields of stemmata (arrows). Abbreviations: ad = abdomen; at = antenna; fe = femur; hc = head capsule; ms = mesothorax; mt = metathorax; pt = prothorax; sy = stylet; ta = tarsus; te = trunk end; ti = tibia.

**Figure 14:**
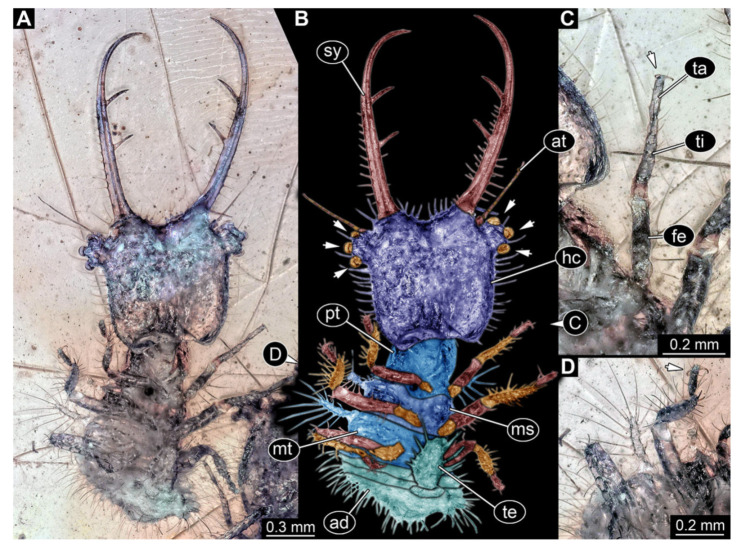
Fossil owllion larva, specimen 3227 (PED 0083a), Cretaceous Kachin amber, Myanmar. (**A**) Overview, ventral. (**B**) Color-marked version of (**A**); arrows point to stemmata. (**C**,**D**) Close-ups on locomotory appendages with claws (arrows). Abbreviations: ad = abdomen; at = antenna; fe = femur; hc = head capsule; ms = mesothorax; mt = metathorax; pt = prothorax; sy = stylet; ta = tarsus; te = trunk end; ti = tibia.

**Figure 15:**
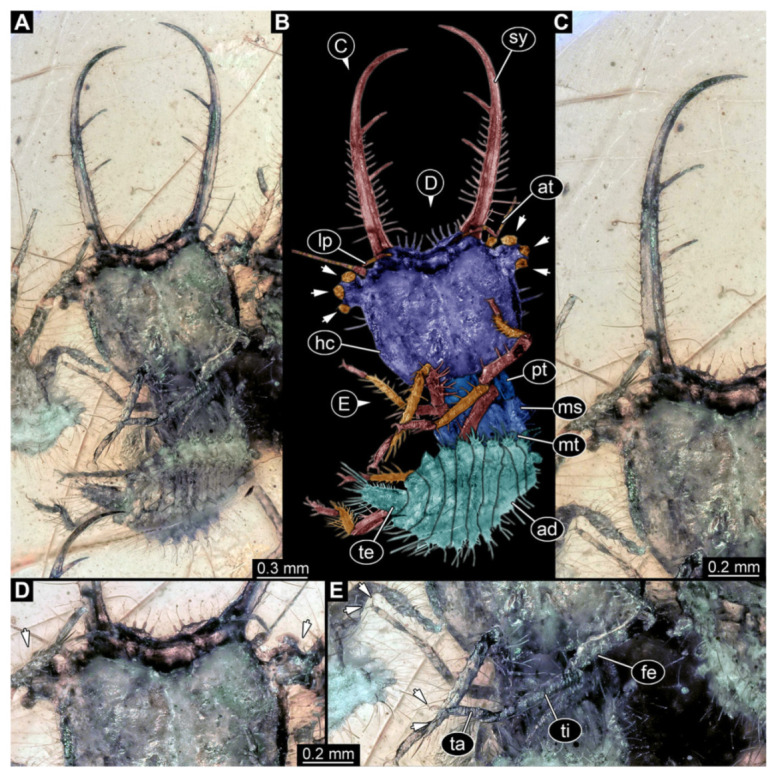
Fossil owllion larva, specimen 3228 (PED 0083b), Cretaceous Kachin amber, Myanmar. (**A**) Overview, ventral. (**B**) Color-marked version of (**A**); arrows point to stemmata. (**C**) Close-up on stylet. (**D**) Close-up on head capsule with fields of stemmata (arrows). (**E**) Close-up on locomotory appendage with claws (arrows). Abbreviations: ad = abdomen; at = antenna; fe = femur; hc = head capsule; lp = labial palp; ms = mesothorax; mt = metathorax; pt = prothorax; sy = stylet; ta = tarsus; te = trunk end; ti = tibia.

**Figure 16:**
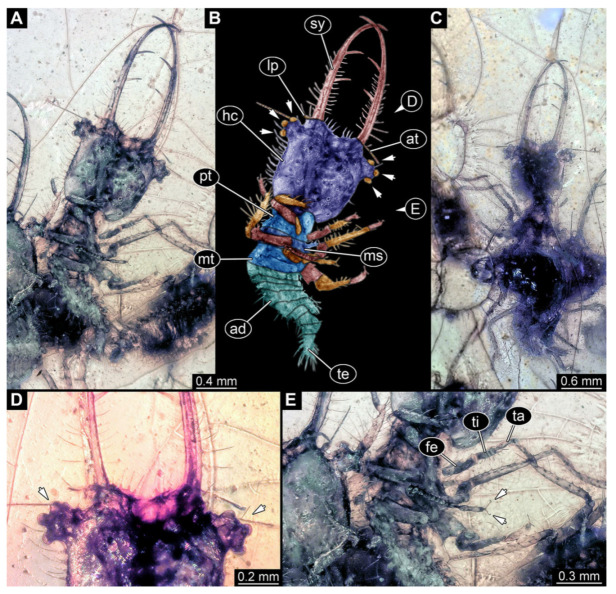
Fossil owllion larva, specimen 3229 (PED 0083c), Cretaceous Kachin amber, Myanmar. (**A**) Overview, ventral. (**B**) Color-marked version of (**A**); arrows point to stemmata. (**C**) Overview, dorsal. (**D**) Close-up on head capsule, with fields of stemmata (arrows). (**E**) Close-up on locomotory appendage with claws (arrows). Abbreviations: ad = abdomen; at = antenna; fe = femur; hc = head capsule; lp = labial palp; ms = mesothorax; mt = metathorax; pt = prothorax; sy = stylet; ta = tarsus; te = trunk end; ti = tibia.

**Figure 17:**
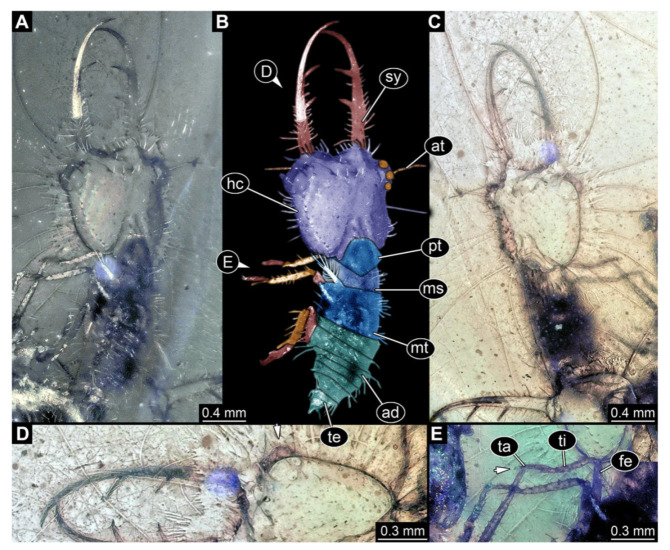
Fossil owllion larva, specimen 3230 (PED 0083d), Cretaceous Kachin amber, Myanmar. (**A**) Overview, dorsal. (**B**) Color-marked version of (**A**). (**C**) Overview, ventral. (**D**) Close-up on head capsule with field of stemmata (arrow). (**E**) Close-up on locomotory appendage with claws (arrow). Abbreviations: ad = abdomen; at = antenna; fe = femur; hc = head capsule; ms = mesothorax; mt = metathorax; pt = prothorax; sy = stylet; ta = tarsus; te = trunk end; ti = tibia.

**Figure 18:**
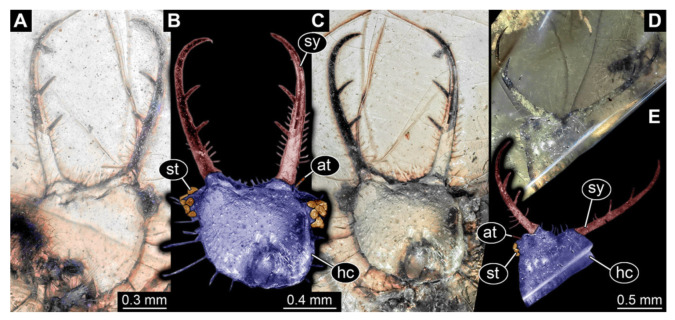
Fossil owllion larvae, Cretaceous Kachin amber, Myanmar. (**A**–**C**) Specimen 3231 (PED 0083e). (**A**) Overview, ventral. (**B**) Color-marked version of (**C**). (**C**) Overview, dorsal. (**D**,**E**) Incomplete specimen (PED 0083f). (**D**) Overview. (**E**) Color-marked version of (**D**) Abbreviations: at = antenna; hc = head capsule; st = stemmata; sy = stylet.

**Figure 19:**
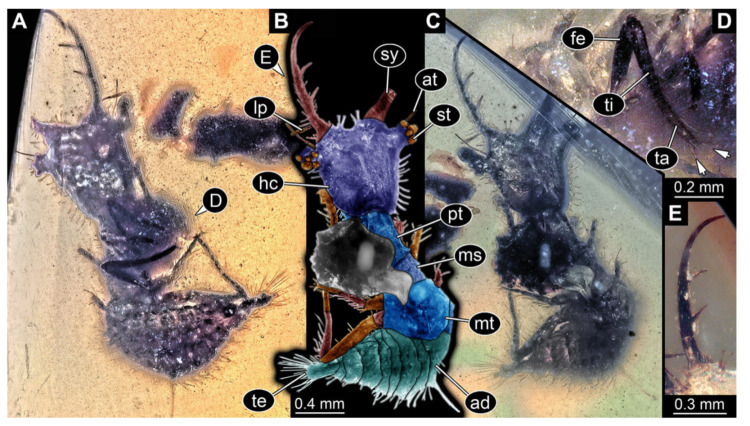
Fossil owllion larva, specimen 3232 (PED 0087), Cretaceous Kachin amber, Myanmar. (**A**) Overview, oblique ventral. (**B**) Color-marked version of (**C**). (**C**) Overview, oblique dorsal. (**D**) Close-up on locomotory appendage with claws (arrows). (**E**) Close-up on stylet. Abbreviations: ad = abdomen; at = antenna; fe = femur; hc = head capsule; lp = labial palp; ms = mesothorax; mt = metathorax; pt = prothorax; st = stemmata; sy = stylet; ta = tarsus; te = trunk end; ti = tibia.

**Figure 20:**
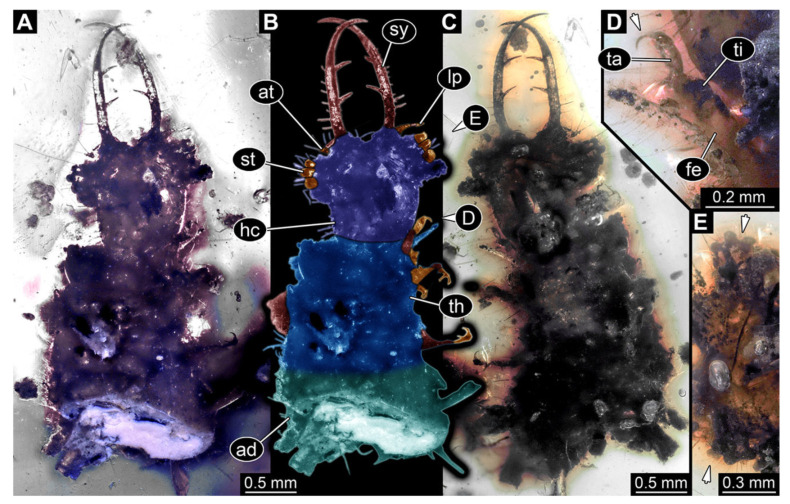
Fossil owllion larva, specimen 3233 (PED 0249), Cretaceous Kachin amber, Myanmar. (**A**) Overview, dorsal. (**B**) Color-marked version of (**A**). (**C**) Overview, ventral. (**D**) Close-up on locomotory appendage with claws (arrow). (**E**) Close-up on head capsule with fields of stemmata (arrows). Abbreviations: ad = abdomen; at = antenna; fe = femur; hc = head capsule; lp = labial palp; st = stemmata; sy = stylet; ta = tarsus; th = thorax; ti = tibia.

**Figure 21:**
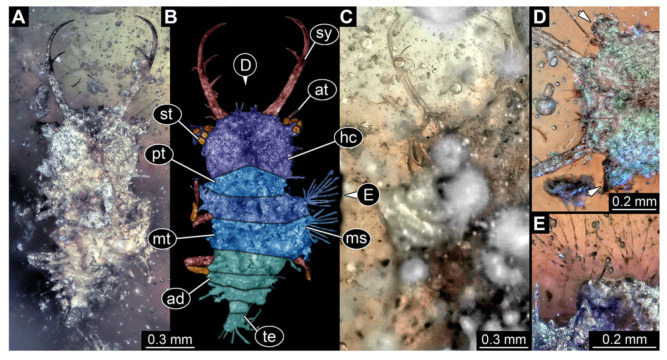
Fossil owllion larva, specimen 3234 (PED 0272), Cretaceous Kachin amber, Myanmar. (**A**) Overview, dorsal. (**B**) Color-marked version of (**A**). (**C**) Overview, ventral. (**D**) Close-up head capsule with fields of stemmata (arrows). (**E**) Close-up on dorsal processes. Abbreviations: ad = abdomen; at = antenna; hc = head capsule; ms = mesothorax; mt = metathorax; pt = prothorax; st = stemmata; sy = stylet; te = trunk end.

**Figure 22:**
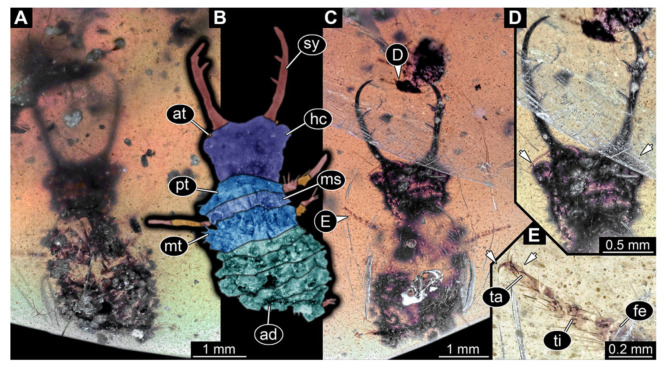
Fossil owllion larva, specimen 3235 (PED 0282), Cretaceous Kachin amber, Myanmar. (**A**) Overview, dorsal. (**B**) Color-marked version of (**A**). (**C**) Overview, ventral. (**D**) Close-up on head capsule with fields of stemmata (arrows). (**E**) Close-up on locomotory appendage with claws (arrows). Abbreviations: ad = abdomen; at = antenna; fe = femur; hc = head capsule; ms = mesothorax; mt = metathorax; pt = prothorax; sy = stylet; ta = tarsus; ti = tibia.

**Figure 23:**
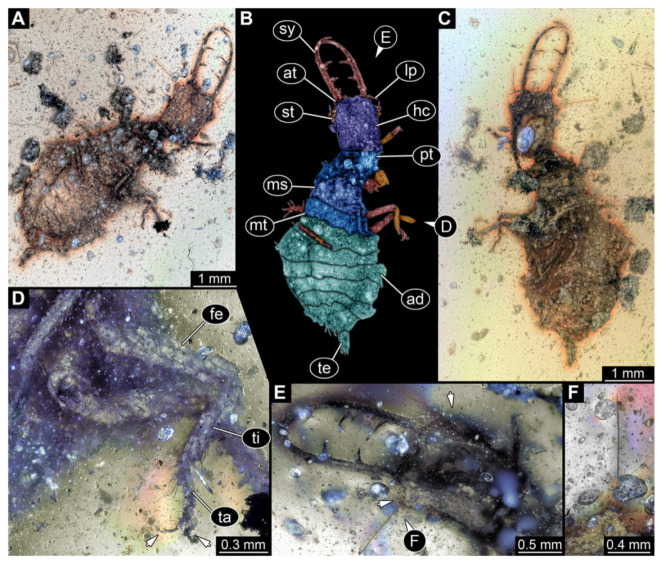
Fossil owllion larva, specimen 3236 (PED 0318), Cretaceous Kachin amber, Myanmar. (**A**) Overview, ventral. (**B**) Color-marked version of (**A**). (**C**) Overview, dorsal. (**D**) Close-up on locomotory appendage with claws (arrows). (**E**) Close-up on head capsule with fields of stemmata (arrows). (**F**) Close-up on antenna. Abbreviations: ad = abdomen; at = antenna; fe = femur; hc = head capsule; lp = labial palp; ms = mesothorax; mt = metathorax; pt = prothorax; st = stemmata; sy = stylet; ta = tarsus; te = trunk end; ti = tibia.

**Figure 24:**
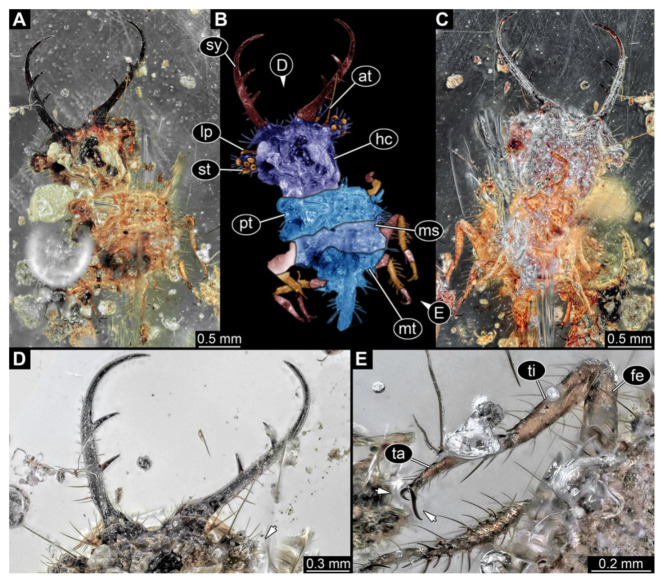
Fossil owllion larva, specimen 3237 (PED 0319), Cretaceous Kachin amber, Myanmar. (**A**) Overview, dorsal. (**B**) Color-marked version of (**A**). (**C**) Overview, ventral. (**D**) Close-up on head capsule with field of stemmata (arrow). (**E**) Close-up on locomotory appendage with claws (arrows). Abbreviations: at = antenna; fe = femur; hc = head capsule; lp = labial palp; ms = mesothorax; mt = metathorax; pt = prothorax; st = stemmata; sy = stylet; ta = tarsus; ti = tibia.

**Figure 25:**
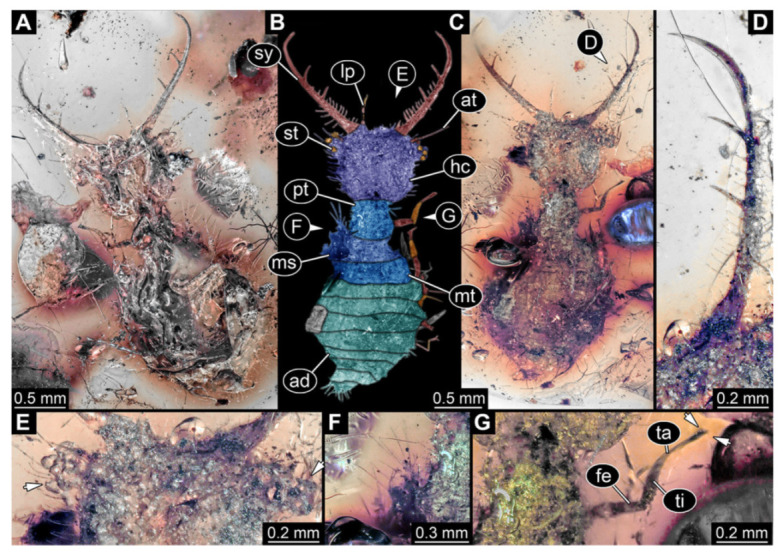
Fossil owllion larva, specimen 3238 (PED 0320), Cretaceous Kachin amber, Myanmar. (**A**) Overview, ventral. (**B**) Color-marked version of (**C**). (**C**) Overview, dorsal. (**D**) Close-up on stylet. (**E**) Close-up on head capsule with fields of stemmata (arrows). (**F**) Close-up on dorsal process. (**G**) Close-up on locomotory appendage with claws (arrows). Abbreviations: ad = abdomen; at = antenna; fe = femur; hc = head capsule; lp = labial palp; ms = mesothorax; mt = metathorax; pt = prothorax; st = stemmata; sy = stylet; ta = tarsus; ti = tibia.

**Figure 26:**
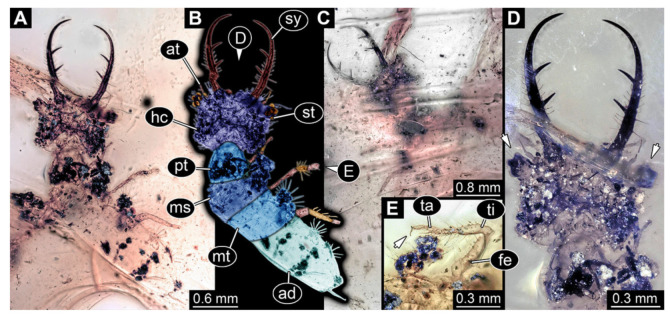
Fossil owllion larva, specimen 3239 (PED 0378), Cretaceous Kachin amber, Myanmar. (**A**) Overview, dorsal. (**B**) Color-marked version of (**A**). (**C**) Overview, ventral. (**D**) Close-up on head capsule with fields of stemmata (arrows). (**E**) Close-up on locomotory appendage with claws (arrow). Abbreviations: ad = abdomen; at = antenna; fe = femur; hc = head capsule; ms = mesothorax; mt = metathorax; pt = prothorax; st = stemmata; sy = stylet; ta = tarsus; ti = tibia.

**Figure 27:**
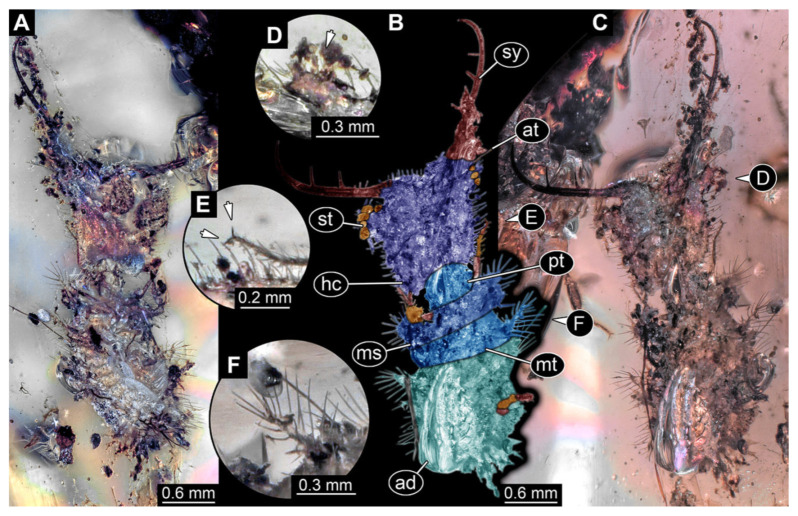
Fossil owllion larva, specimen 3240 (PED 0520), Cretaceous Kachin amber, Myanmar. (**A**) Overview, ventral. (**B**) Color-marked version of (**C**). (**C**) Overview, dorsal. (**D**) Close-up on field of stemmata (arrow). (**E**) Close-up on locomotory appendage with claws (arrows). (**F**) Close-up on dorsal process. Abbreviations: ad = abdomen; at = antenna; hc = head capsule; ms = mesothorax; mt = metathorax; pt = prothorax; st = stemmata; sy = stylet.

**Figure 28:**
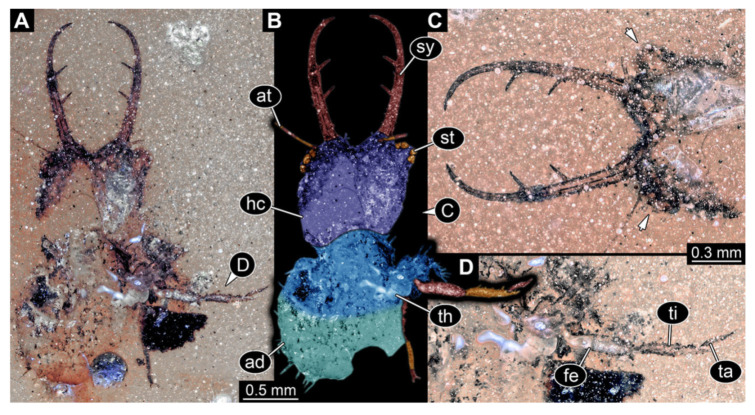
Fossil owllion larva, specimen 3241 (PED 0563), Cretaceous Kachin amber, Myanmar. (**A**) Overview, dorsal. (**B**) Color-marked version of (**A**). (**C**) Close-up on head capsule with fields of stemmata (arrows). (**D**) Close-up on locomotory appendage. Abbreviations: ad = abdomen; at = antenna; fe = femur; hc = head capsule; st = stemmata; sy = stylet; ta = tarsus; th = thorax; ti = tibia.

**Figure 29:**
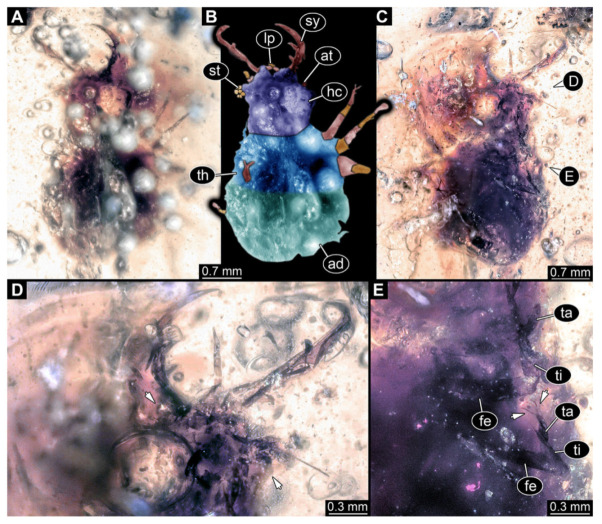
Fossil owllion larva, specimen 3242 (PED 0575), Cretaceous Kachin amber, Myanmar. (**A**) Overview, dorsal. (**B**) Color-marked version of (**A**). (**C**) Overview, ventral. (**D**) Close-up on head capsule with fields of stemmata (arrows). (**E**) Close-up on locomotory appendages with claws (arrows). Abbreviations: ad = abdomen; at = antenna; fe = femur; hc = head capsule; lp = labial palp; st = stemmata; sy = stylet; ta = tarsus; th = thorax; ti = tibia.

**Figure 30:**
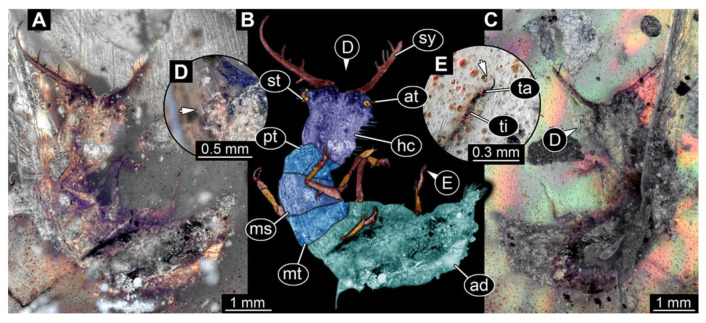
Fossil owllion larva, specimen 3243 (PED 0583), Cretaceous Kachin amber, Myanmar. (**A**) Overview, ventral. (**B**) Color-marked version of (**A**). (**C**) Overview, dorsal. (**D**) Close-up on head capsule with field of stemmata (arrows). (**E**) Close-up on locomotory appendage with claws (arrow). Abbreviations: ad = abdomen; at = antenna; hc = head capsule; ms = mesothorax; mt = metathorax; pt = prothorax; st = stemmata; sy = stylet; ta = tarsus; ti = tibia.

**Figure 31:**
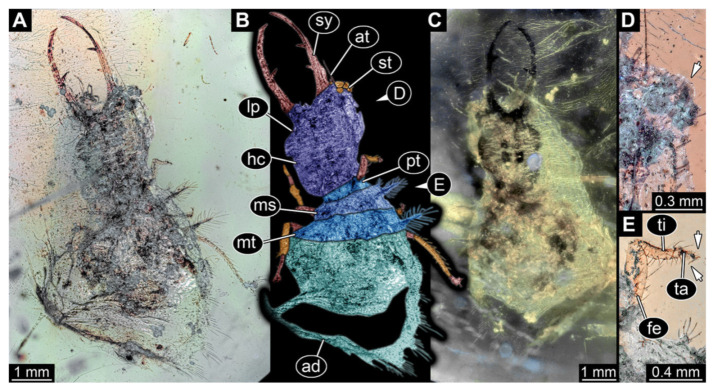
Fossil owllion larva, specimen 3244 (PED 0684), Cretaceous Kachin amber, Myanmar. (**A**) Overview, dorsal. (**B**) Color-marked version of (**A**). (**C**) Overview, ventral. (**D**) Close-up on head capsule with field of stemmata (arrow). (**E**) Close-up on locomotory appendage with claws (arrows). Abbreviations: ad = abdomen; at = antenna; fe = femur; hc = head capsule; lp = labial palp; ms = mesothorax; mt = metathorax; pt = prothorax; st = stemmata; sy = stylet; ta = tarsus; ti = tibia.

**Figure 32:**
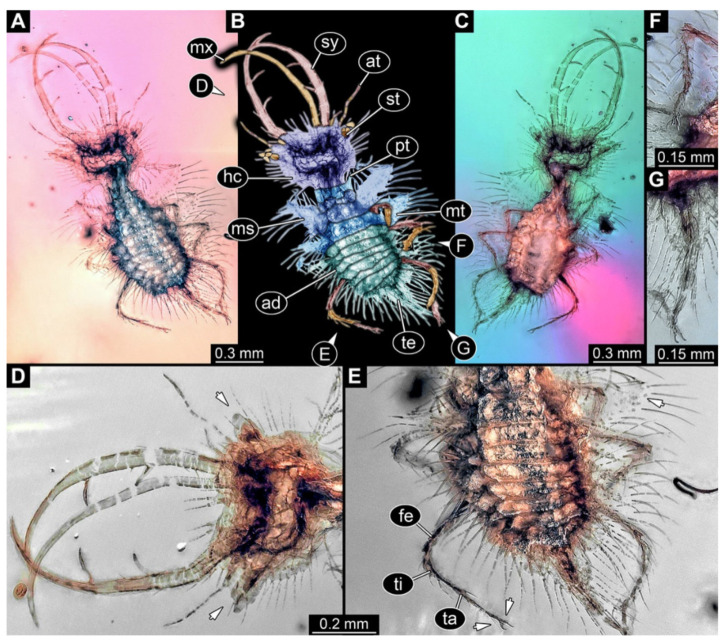
Fossil owllion larva, specimen 3245 (PED 0944), Cretaceous Kachin amber, Myanmar. (**A**) Overview, dorsal. (**B**) Color-marked version of (**A**). (**C**) Overview, ventral. (**D**) Close-up on head capsule with fields of stemmata (arrows). (**E**) Close-up on trunk with locomotory appendages with claws (left arrows) and dorsal process (right arrow). (**F**,**G**) Close-ups on locomotory appendages. Abbreviations: ad = abdomen; at = antenna; fe = femur; hc = head capsule; ms = mesothorax; mt = metathorax; mx = maxilla; pt = prothorax; st = stemmata; sy = stylet; ta = tarsus; te = trunk end; ti = tibia.

**Figure 33:**
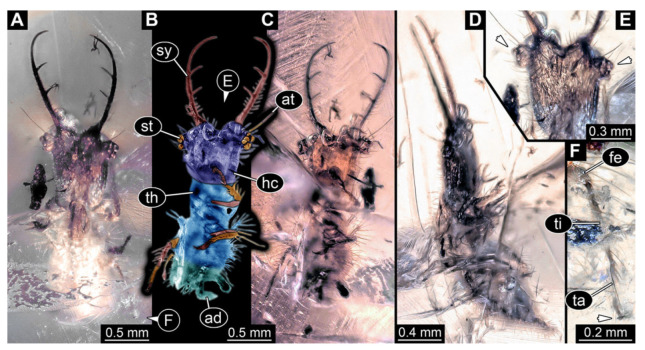
Fossil owllion larva, specimen 3246 (PED 0975), Cretaceous Kachin amber, Myanmar. (**A**) Overview, dorsal. (**B**) Color-marked version of (**C**). (**C**) Overview, ventral. (**D**) Overview, lateral. (**E**) Close-up on head capsule with fields of stemmata (arrows). (**F**) Close-up on trunk with locomotory appendage with claws (arrow). Abbreviations: ad = abdomen; at = antenna; fe = femur; hc = head capsule; st = stemmata; sy = stylet; ta = tarsus; th = thorax; ti = tibia.

**Figure 34:**
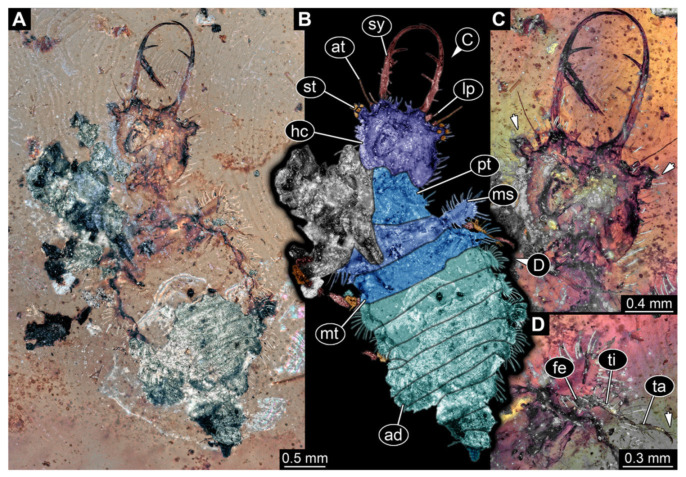
Fossil owllion larva, specimen 3247 (PED 1047), Cretaceous Kachin amber, Myanmar. (**A**) Overview, dorsal. (**B**) Color-marked version of (**A**). (**C**) Close-up on head capsule with fields of stemmata (arrows). (**D**) Close-up on trunk with locomotory appendages with claws (arrow). Abbreviations: ad = abdomen; at = antenna; fe = femur; hc = head capsule; lp = labial palp; ms = mesothorax; mt = metathorax; pt = prothorax; st = stemmata; sy = stylet; ta = tarsus; ti = tibia.

**Figure 35:**
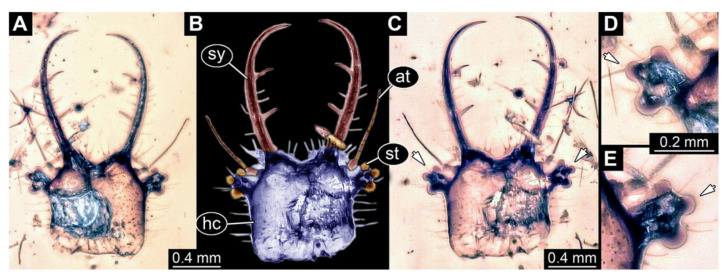
Fossil owllion larva, specimen 3248 (PED 1206), Cretaceous Kachin amber, Myanmar. (**A**) Overview, one side. (**B**) Color-marked version of (**C**). (**C**) Overview, other side. (**D**,**E**) Close-ups on fields of stemmata (arrows). Abbreviations: at = antenna; hc = head capsule; st = stemmata; sy = stylet.

**Figure 36:**
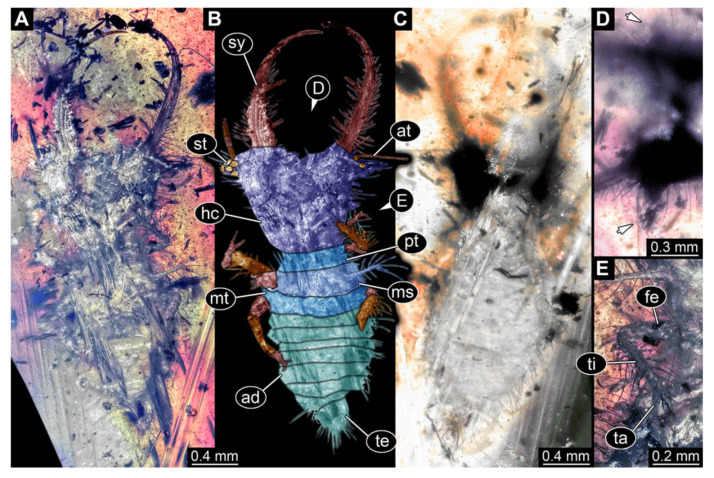
Fossil owllion larva, specimen 3249 (Weiterschan BuB 23), Cretaceous Kachin amber, Myanmar. (**A**) Overview, ventral. (**B**) Color-marked version of (**A**). (**C**) Overview, dorsal. (**D**) Close-up on head capsule with fields of stemmata (arrows). (**E**) Close-up on trunk with locomotory appendages. Abbreviations: ad = abdomen; at = antenna; fe = femur; hc = head capsule; ms = mesothorax; mt = metathorax; pt = prothorax; st = stemmata; sy = stylet; ta = tarsus; te = trunk end; ti = tibia.

## Data Availability

All data from this study are available in this paper and the associated papers.

## Supplementary Materials

The following supporting information can be downloaded at: https://www.mdpi.com/article/10.3390/insects13070587/s1, File S1: Information on the specimens investigated in this study. Abbreviations: Eoc = Eocene; K = Cretaceous; Mio = Miocene. For institutional abbreviations, see Material and Methods section and references provided in the table; File S2: Additional references for table in File S1 not referred to in main text [103,104,105,106,107,108,109,110,111,112,113,114,115,116,117,118,119,120,121,122,123,124,125]; File S3: Results of the principal component analysis; File S4: Graphical representation of the factor loadings of the principal component analysis; File S5: Files resulting from the shape analysis, including chain codes, aligned shapes, and principal component analysis; Figure 1, Figure 2, Figure 3, Figure 4, Figure 5, Figure 6, Figure 7, Figure 8, Figure 9, Figure 10, Figure 11, Figure 12, Figure 13, Figure 14, Figure 15, Figure 16, Figure 17, Figure 18, Figure 19, Figure 20, Figure 21, Figure 22, Figure 23, Figure 24, Figure 25, Figure 26, Figure 27, Figure 28, Figure 29, Figure 30, Figure 31, Figure 32, Figure 33, Figure 34, Figure 35, Figure 36, Figure 37, Figure 38, Figure 39 and Figure 40 of high resolution are available in the Supplementary Materials.
