# Supplementary figures and images for: The Morphological Diversity of Antlion Larvae and Their Closest Relatives over 100 Million Years
Source: Insects. 2022 Jun 27;13(7):587. doi: 10.3390/insects13070587 (PMC9316203; doi:10.3390/insects13070587)

-2S.D.      Mean      +2S.D.

PC1

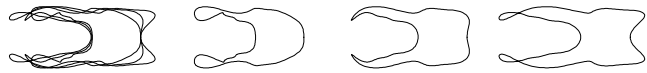

PC2

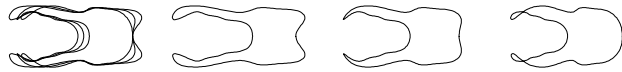

PC3

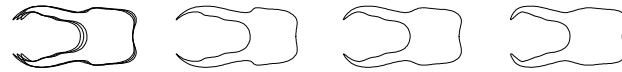

Supplement: Supplementary file 1 [file insects-13-00587-s001.zip › insects-1777049-supplementary/Supplement/File_S4_owllions_factor_loadings.pdf]
